# Recent Advances in 1D Nanostructure Assembly and Direct Integration Methods for Device Applications

**DOI:** 10.1002/smtd.202400474

**Published:** 2024-08-07

**Authors:** Incheol Cho, Jiwoo Ko, Dionisio Del Orbe Henriquez, Daejong Yang, Inkyu Park

**Affiliations:** ^1^ Department of Mechanical Engineering Korea Advanced Institute of Science and Technology (KAIST) Daejeon 34141 Republic of Korea; ^2^ Department of Mechanical and Automotive Engineering Kongju National University Cheonan 31080 Republic of Korea; ^3^ Department of Future Convergence Engineering Kongju National University Cheonan 31080 Republic of Korea

**Keywords:** assembly, electrodes, energy harvesters, integration, nanotubes, nanowires, sensors

## Abstract

In recent years, 1D nanostructure‐based devices have achieved widespread usage in various fields, such as sensors, energy harvesters, transistors, and electrodes owing to their exceptional and distinct properties. The pioneering work of Dr. R. S. Wagner at Bell Laboratories in 1964 introduced the vapor–liquid–solid (VLS) process, a powerful synthesis method. Since then, numerous synthesis techniques, including sol–gel, hydrothermal, chemical vapor deposition (CVD), physical vapor deposition (PVD), and more, have been developed. These methods have enabled researchers to effectively control the shape (length and diameter) and material properties of nanowires. However, it was only about two decades ago that nanowires started to be widely utilized as key components in functional devices, primarily due to the lack of proper integration methods. Although dozens of integration techniques have been developed, none have emerged as a predominant choice, with each method presenting its own set of advantages and limitations. Therefore, this work aims to categorize these methods based on their working principles and provide a comprehensive summary of their pros and cons. Additionally, state‐of‐the‐art devices that capitalize on the integration of 1D nanomaterials are introduced.

## Graphical abstract

Various assembly and integration methods are presented for harnessing high‐performance nanomaterials in devices. These methods, including deposition, contact printing, microfluidic force assembly, electromagnetic force assembly, optical assembly, and substrate deformation, are categorized, with their respective advantages and disadvantages outlined. Additionally, direct integration approaches, categorized as patterning and local heating, are introduced along with their pros and cons.

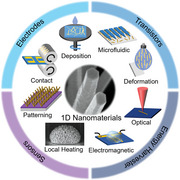

## 1 Introduction

Over the past two decades, extensive research has been conducted on 1D nanostructures due to their exceptional properties and numerous advantages.^[^ ^1^, ^2^, ^3^, ^4^ ^]^ In terms of mechanical properties, nanowires (NWs) provide high ultimate strengths and Young's moduli owing to their low density of defects and grain size effects.^[^ ^5^, ^6^, ^7^ ^]^ Electrically, they show high and quantized thermal and electrical conductivities due to their low density of defects and dimensions below the mean free paths of phonons and electrons.^[^ ^8^, ^9^, ^10^ ^]^ Moreover, they possess unique characteristics that are challenging to achieve in bulk materials, such as tunable band gaps and extremely high surface‐to‐volume ratios.^[^ ^11^, ^12^ ^]^ These distinctive properties have facilitated the widespread utilization of 1D nanostructures in various electrical and mechanical devices, including field‐effect transistors (FETs), biological/chemical/photonic sensors, energy harvesters/storage devices, and optical components.^[^ ^13^ ^]^

There are a lot of research to synthesize 1D nanostructures and their approaches can be categorized into two types: top‐down and bottom‐up methods.^[^ ^14^ ^]^ Top‐down methods start from bulk materials and physically cut or chemically etch them out to designed shapes, while bottom‐up methods assemble molecules from precursors into chemically synthesized nanostructures. Bottom‐up methods can be further classified as sol–gel, hydrothermal, chemical vapor deposition (CVD), physical vapor deposition (PVD), and other techniques, based on the source materials and phase transition processes.^[^ ^14^ ^]^

**Figure 1:**
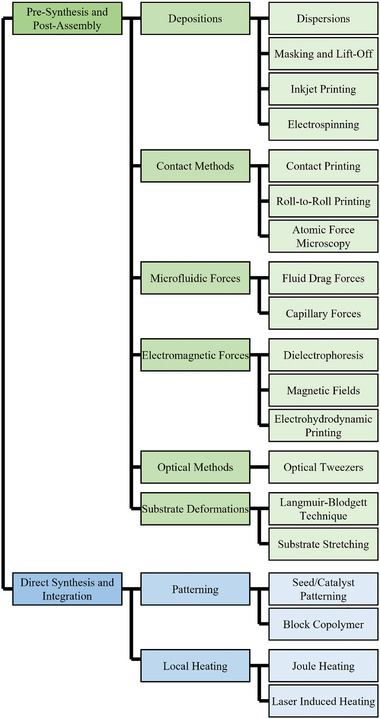
Classification of 1D nanostructures assembly and integration methods.

To utilize these nanostructures in devices, they must be connected to electrodes or assembled at specific positions. Therefore, numerous methods have been developed for the controlled assembly and integration of 1D nanostructures. In this article, we categorize these methods into two groups: 1) pre‐synthesis and post‐assembly methods, and 2) direct synthesis and in‐situ integration methods. **Figure** **1** illustrates the classification of pre‐synthesis and post‐integration methods based on integration techniques, including deposition, contact printing, microfluidic force assembly, electromagnetic force assembly, optical assembly, and substrate deformation. Additionally, we classify the direct synthesis and in‐situ integration methods based on fabrication techniques involving patterning and locally focused energy.

Furthermore, while several review papers have addressed the synthesis of 1D nanostructures, fabrication of nanodevices, and their applications in various fields, this paper aims to focus on the integration methods of 1D nanostructures, which has received limited attention in previous review papers. Therefore, we believe that this paper will provide useful information, deep insights, and creative inspiration to researchers on the integration of 1D nanostructures toward advanced functional nanodevices applications.

## 2 Pre‐Synthesis and Post‐Assembly of 1D Nanostructures

For nanomaterials to function as components in devices, they need to be precisely positioned and electrically or mechanically connected to other parts. However, selectively synthesizing nanomaterials at specific positions is challenging. Therefore, in the early stages of nanodevice research, pre‐synthesized nanostructures were commonly transferred to the desired locations,^[^ ^15^ ^]^ and this method remains a fundamental aspect of nanodevice fabrication to this day. In this approach, nanodevices are typically fabricated through the following steps: a large volume of nanomaterials is produced using wet or vaporized chemical reactions such as sol–gel, hydrothermal, CVD, and PVD.^[^ ^16^, ^17^, ^18^, ^19^, ^20^, ^21^, ^22^, ^23^ ^]^ Subsequently, these nanomaterials are harvested and suspended in solutions like ethanol, methanol, ethylene glycol, or water. The suspended nanomaterials are then dispersed onto the target substrate using a variety of techniques, which are described in detail in the following subsections. To fabricate sophisticated and high‐performance nanodevices, numerous techniques for the alignment and assembly of nanomaterials have been developed. In this section, we categorize these techniques based on their underlying physical principles and provide a summary of each method.

### 2.1 Assembly of 1D Nanostructures by Deposition

Deposition is the most basic and widely used method for fabricating nanodevices. In the early stages, nanomaterials were dispersed onto pre‐fabricated electrodes.^[^ ^24^ ^]^ However, this approach resulted in significant device‐to‐device variation and limitations in downscaling, thereby hindering the full utilization of the advantages provided by nanodevices. To overcome these challenges, researchers have explored various techniques for pattern formation, including conventional photolithography, inkjet printing, and electrospinning.^[^ ^25^, ^26^, ^27^, ^28^, ^29^, ^30^, ^31^ ^]^ These methods provide more precise control over the placement of 1D nanostructures, facilitating the fabrication of nanodevices with improved performance and reduced deviation.

#### 2.1.1 Dispersion

Dispersion is the simplest method for depositing NWs onto substrates, and it involves popular methods such as drop casting,^[^ ^21^ ^]^ spin coating,^[^ ^25^, ^27^, ^32^ ^]^ and spray coating.^[^ ^33^ ^]^ In drop casting, pre‐synthesized nanomaterials are suspended in liquid solutions and deposited onto the target substrate by drop casting the solution.^[^ ^24^ ^]^ Afterward, the solution is then evaporated, leaving the nanostructures attached to the substrate. While this method is straightforward and allows rough control over the density of nanostructures, achieving precise integration can be challenging. To achieve more uniform distribution and control over density, researchers have utilized spray coating and spin coating methods.^[^ ^34^ ^]^

**Figure 2:**
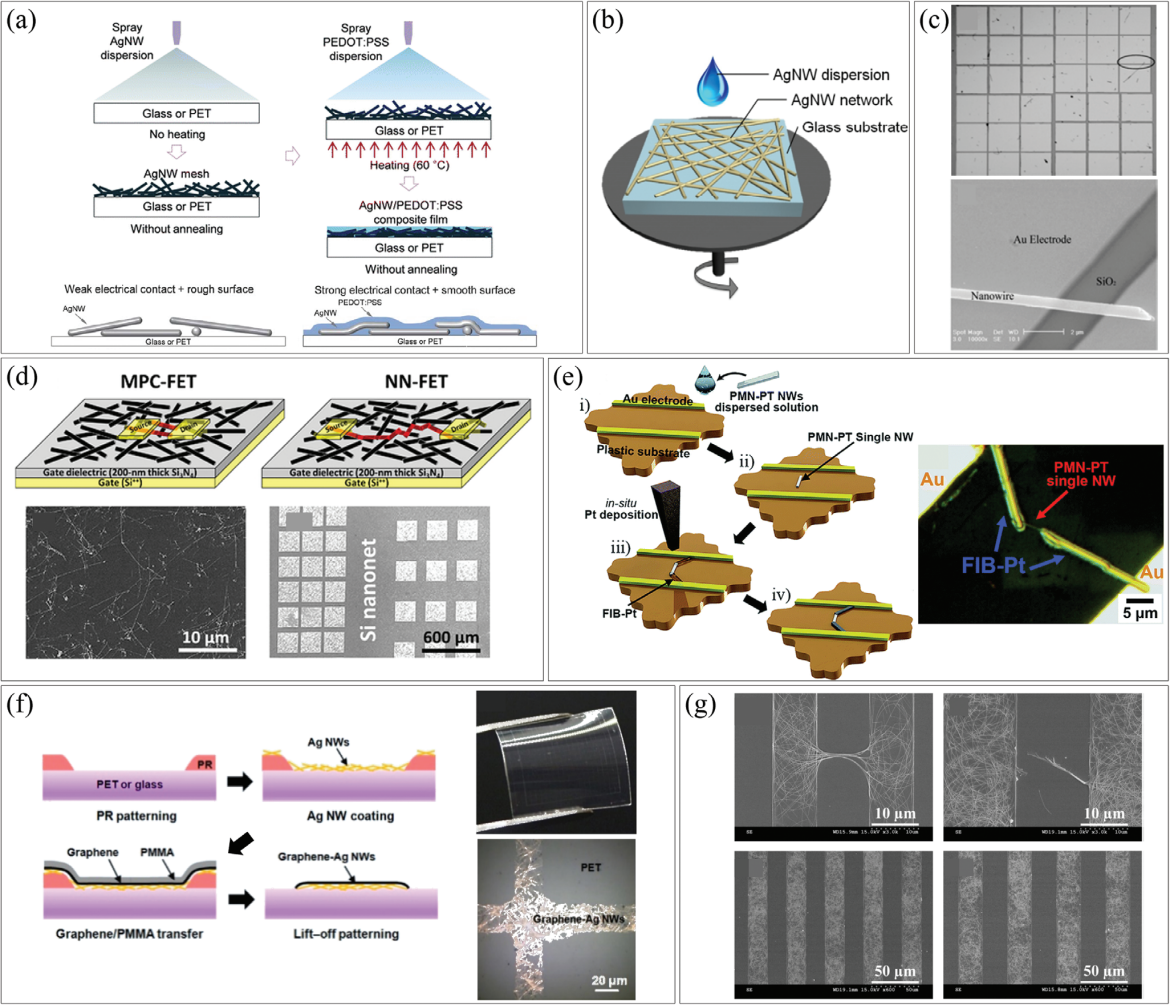
Assembly of 1D nanostructures by dispersion. a) Fabrication process of AgNW‐PEDOT:PSS composite electrodes using spray coating. Reproduced with permission.^[^ ^33^ ^]^ Copyright 2013, Royal Society of Chemistry. b) Schematic illustration of the spin coating process of AgNW network on a glass substrate. Reproduced with permission.^[^ ^32^ ^]^ Copyright 2013, Optica Publishing Group. c) SEM images showing the contact between Cu‐TCNQ NW and Au pads via dispersion. Reproduced with permission.^[^ ^35^ ^]^ Copyright 2009, Published by Springer Nature Limited. d) (top) Schematic illustration and (bottom) SEM images of SiNW channels fabricated by dispersion and filtration. Reproduced with permission.^[^ ^36^ ^]^ Copyright 2020, AIP Publishing. e) (left) Fabrication process combining PMN–PT NW dispersion and Pt electrode deposition, and (right) SEM image of SiNW connected to Pt electrodes. Reproduced with permission.^[^ ^37^ ^]^ Copyright 2017, Royal Society of Chemistry. Assembly of 1D nanostructures by masking and lift‐off. f) (left) Schematic illustration of the lift‐off patterning procedure of graphene‐Ag NWs, and (right) photograph and optical images of 20 µm grid patterns of graphene‐Ag NWs on PET substrates. Reproduced with permission.^[^ ^38^ ^]^ Copyright 2017, American Chemical Society. g) SEM images of the patterned AgNW films with widths of (top left) 10 µm, (top right) 15 µm, (bottom left) 20 µm and (bottom right) 25 µm. Reproduced with permission.^[^ ^27^ ^]^ Copyright 2015, Elsevier.

Spray coating is a method of spraying a nanomaterial‐suspended solution onto a substrate by loading it in a carrier gas. It is suitable for printing dense nanomaterials and can be applied to relatively large areas with moderate uniformity. However, nozzle clogging may occur if the solution viscosity is too high or if the NWs are long. Spray coating has been utilized in large‐area film manufacturing processes, such as roll‐to‐roll coating. For example, Choi et al. used two steps of spray coating to fabricate high‐efficiency flexible optoelectronic devices, as depicted in **Figure** **2**a.^[^ ^33^ ^]^ First, silver (Ag) NWs were spray‐coated as electrodes, followed by the spray coating of poly(3,4‐ethylenedioxythiophene)‐poly(styrenesulfonate) (PEDOT:PSS) with dimethyl sulfoxide (DMSO) to enhance electrical contact and reduce surface roughness. During the second spray coating process, the AgNW mesh was heated to 60 °C which promoted the evaporation of the solvent. This heating combined with spray coating processes is expected to be suitable for the continuous production of flexible devices by combining with roll‐to‐roll processes.

In the spin coating process, a substrate is placed onto a rotation disk, and a liquid suspension containing a functional nanomaterial is applied. The substrate is then rapidly rotated, and the solvent is evaporated. The film thickness can be controlled by the suspension viscosity and substrate rotation speed. However, when the viscosity is low, defects such as the coffee‐ring effect may occur during solvent evaporation, which limits control over the density and thickness of the NWs. Xie et al. utilized spin coating of AgNWs to fabricate large‐sized and highly uniform silicon‐based solar cells.^[^ ^32^ ^]^ They optimized a two‐step annealing process to obtain transparent electrodes with superior electrical and optical properties. This process offered the advantages of easy processing and high uniformity and they were able to obtain uniform and highly conductive films with a variation of less than 10% (Figure 2b).

Dispersion methods are effective for distributing NWs over a large area and typically use high‐density solutions. However, in some cases, researchers employ dispersion methods for individual NW devices. They have dispersed a very low concentration of NW‐suspended solution on a substrate to avoid contact between NWs. For instance, Zheng et al. prepared a patterned substrate with a square electrode array and dispersed copper 7,7,8,8‐Tetracyanoquinodimethane (Cu‐TCNQ) NWs.^[^ ^35^ ^]^ They selected individual NWs with both ends in contact with electrodes and conducted experiments to analyze the electrical characteristics of the NWs (Figure 2c). Hien et al. reversed the process sequence, dispersing copper oxide (CuO) NWs onto a substrate and then depositing gold (Au) electrodes using conventional photolithography and a lift‐off process.^[^ ^25^ ^]^ A single NW connected to a pair of electrodes was selected and used as a FET. Legallais et al. proposed a method to obtain more versatile and superior NW characteristics.^[^ ^36^ ^]^ They dispersed silicon (Si) NWs and filtered them to make a thin film structure, which was then transferred onto a substrate. By using the spacing of the pre‐deposited electrodes, FETs with a single NW channel and other FETs composed of an NW network were fabricated (Figure 2d). In the latter case, the junctions between NWs played an important role, resulting in significantly lower off‐state current and improved subthreshold slope of the FET. Moorthy et al. presented a more advanced technique.^[^ ^37^ ^]^ They first dispersed 0.65Pb(Mg_1/3_Nb_2/3_)O_3_–0.35PbTiO_3_ (PMN–PT) NWs and then connected the ends of the NWs to the electrodes using a metal deposition process involving focused ion beam (FIB) (Figure 2e). These three methods have limited productivity and practicality challenges since they require careful selection of suitable NWs and reliable connections between the NWs and electrodes.

While dispersion methods are effective in distributing NWs over a large area, achieving precise control over individual NWs or all NWs is challenging. Therefore, to ensure the desired functionality and performance of nanodevices, it is imperative to employ additional processes that can align individual NWs.

#### 2.1.2 Masking and Lift‐Off

Although the aforementioned dispersion methods are simple, they are primarily suitable for depositing nanomaterials over large areas due to limitations in patterning resolution. To minimize material waste, utilize space efficiently, and fabricate more sophisticated devices with high resolution, several methods have been developed for patterning nanomaterials in specific desired areas.

In masking and lift‐off methods, a predefined pattern is created on a substrate, and functional materials are deposited onto the substrate. Undesired layers are then removed using a solvent. For instance, Trung et al. coated pre‐synthesized AgNWs, graphene film, and poly(Methyl Methacrylate) (PMMA) onto a photoresist (PR) pattern, and the materials on the PR were removed by the lift‐off process (Figure 2f).^[^ ^38^ ^]^ Similarly, Kim et al. utilized a lift‐off process to fabricate AgNW/PEDOT:PSS patterns (Figure 2g).^[^ ^27^ ^]^

Compared to the previously mentioned dispersion methods, these techniques enable the creation of finer patterns with dimensions in the micrometer range. However, since they are also based on dispersion, there is no inherent difference in device characteristics between the dispersion method and the masking and lift‐off method. In subsequent sections, we introduce direct patterning methods that allow for precise application of nanostructures in designated areas.

#### 2.1.3 Inkjet Printing

The principle of inkjet printing for nanostructure is the same as printing documents on paper with the difference being the type of ink used.^[^ ^39^ ^]^ Instead of colored ink, solutions containing suspended nanostructures are used. These nanostructures encompass metals, semiconductors, graphene (including graphene oxide), and polymers in the form of nanoparticles (NPs), NWs, nanotubes (NTs), and nanosheets.^[^ ^39^ ^]^ The pattern resolution of this method is determined by the nozzle size, allowing for the printing of patterns ranging from several hundreds of nanometers to several millimeters in size on large‐scale substrates.^[^ ^40^ ^]^ This method does not require pre‐designed masks and can be operated at room temperature and normal atmospheric pressure. These mild fabrication conditions enable printing on plastic substrates, making it suitable for flexible devices.^[^ ^41^ ^]^

**Figure 3:**
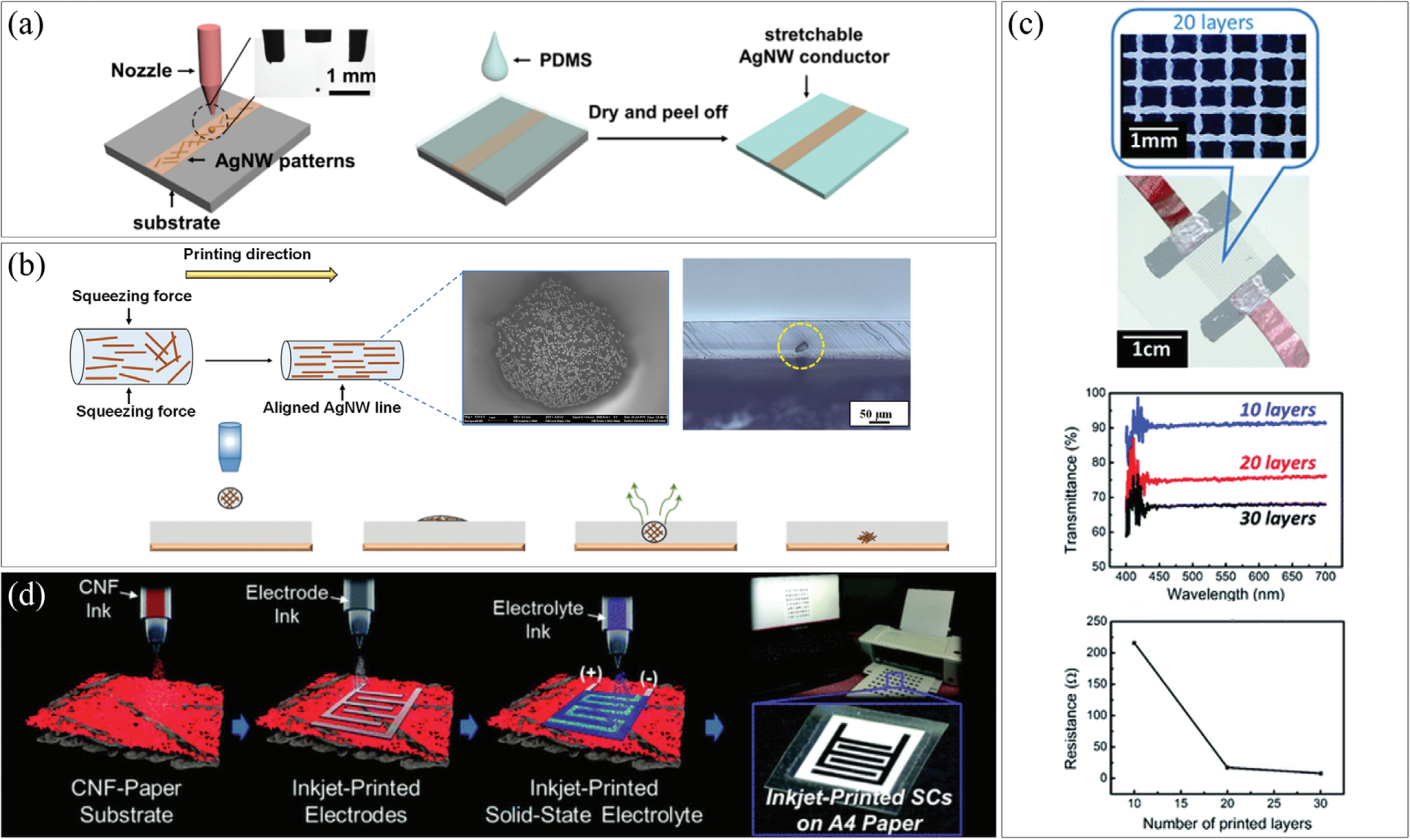
Assembly of 1D nanostructures via inkjet printing. a) Fabrication process of AgNW pattern combined with a stretchable polymer substrate by using inkjet printing. Reproduced with permission.^[^ ^28^ ^]^ Copyright 2018, American Chemical Society. b) Fabrication process of AgNWs embedded polymer film using inkjet printing and images of the substrate. Reproduced with permission.^[^ ^42^ ^]^ Copyright 2020, American Chemical Society. c) (top) Optical microscope image of the printed AgNW mesh on glass and (bottom) variation of transmittance and resistance with the number of AgNW mesh layers. Reproduced with permission.^[^ ^29^ ^]^ Copyright 2015, Royal Society of Chemistry. d) Fabrication procedure of the inkjet‐printed solid‐state capacitors. Reproduced with permission.^[^ ^30^ ^]^ Copyright 2016, The Royal Society of Chemistry.

For example, Huang et al. demonstrated the fabrication of a stretchable heater by combining an inkjet‐printed AgNW pattern with spin‐coated polydimethylsiloxane (PDMS) as shown in **Figure** **3**a.^[^ ^28^ ^]^ This work showed the potential of inkjet printing for large‐scale and flexible electronics. In another approach, Al‐Milaji et al. utilized inkjet printing to fabricate an AgNWs‐embedded polymer film, as shown in Figure 3b.^[^ ^42^ ^]^ They printed AgNWs onto an uncured PDMS film, and as the solvent in the ink evaporated, the AgNWs agglomerated and became immersed in the PDMS film. The depth of the NWs was controlled by adjusting the sinking time, and the PDMS was subsequently cured to secure them in place. Wang et al. utilized AgNW ink to print a mesh grid structure, resulting in a heater with high electrical conductivity and 90% transparency, as shown in Figure 3c.^[^ ^29^ ^]^ The conductivity and transparency of the mesh were adjustable by controlling the diameter and density of the NWs. In inkjet printing, the ejection of ink solution from the nozzles is a critical aspect. Nozzle clogging is a major obstacle to productivity and resolution. Finn et al. suggested a way to solve this problem using an optimized isopropyl alcohol‐diethylene glycol solution, enabling the fabrication AgNW patterns with high accuracy and resolution of 100 µm.^[^ ^39^ ^]^ Another method involves using high‐boiling‐point solutions to print nanomaterials at high temperatures with low viscosity.^[^ ^43^ ^]^ Nevertheless, there is an inherent conflict between the length of the nanostructure and nozzle clogging.^[^ ^44^ ^]^ Choi et al. successfully fabricated solid‐state flexible supercapacitors on a conventional A4 paper substrate using a commercial desktop inkjet printer, as depicted in Figure 3d.^[^ ^30^ ^]^ The supercapacitors were composed of cellulose nanofibril‐mediated nanomat, carbon nanotubes (CNTs)‐assisted photonic interwelded AgNWs, and 1‐butyl‐3‐methylimidazolium tetrafluoroborate and trimethylolpropane ethoxylate triacrylate ([BMIM][BF_4_]/ETPTA) mixture electrolytes, all of which were inkjet‐printed. This printed supercapacitor closely resembled actual inkjet‐printed documents, exhibiting similar resolution.

#### 2.1.4 Electrospinning

**Figure 4:**
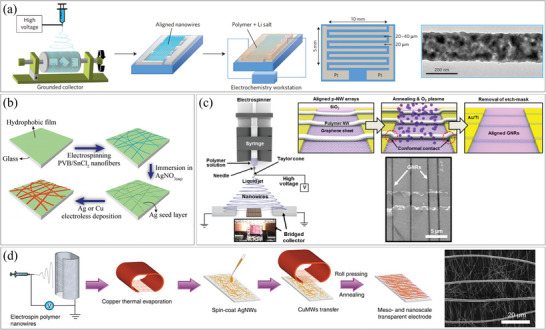
Assembly of 1D nanostructures via electrospinning. a) (left) Synthesis procedure for the composite polymer electrolyte with aligned NW, (middle) schematic illustration of interdigitated Pt electrodes, and (right) transmission electron microscopy (TEM) images of the LLTO NWs. Reproduced with permission.^[^ ^46^ ^]^ Copyright 2017, Springer Nature Limited. b) Fabrication process of transparent electrodes using a combined electrospinning and electroless deposition method. Reproduced with permission.^[^ ^47^ ^]^ Copyright 2014, American Chemical Society. c) (left) Schematic illustration of electrospinning setup, (right top) fabrication process consisting of electrospinning and etching, and (right bottom) SEM image of graphene nanoribbons. Reproduced with permission.^[^ ^48^ ^]^ Copyright 2021, The Authors, Published by MDPI. d) Fabrication procedure for meso‐ and nanoscale transparent electrode and SEM image of the electrode. Reproduced with permission.^[^ ^49^ ^]^ Copyright 2013, Springer Nature Limited.

Electrospinning is a method used to synthesize nanofibers by applying an electric field. An electrical bias is applied between the nozzle and the target substrate, which discharges polymer solution or melted polymer containing nanostructures. The size of the printed fibers is determined by the applied voltage, needle size, and electric and fluidic properties of the precursor materials.^[^ ^45^ ^]^ Electrospinning can produce nanofibers smaller than the diameter of the nozzle and longer compared to inkjet printing. However, precise control over the printing position can be challenging. Nanofibers are sometimes used in their randomly dispersed form (**Figure** **4**b), but they are more commonly used in aligned forms. Nanofibers can be mechanically aligned by rotating a grounded collector, as demonstrated in Figure 4a, or they can be aligned electrically using an electric field, as shown in Figures 4c,d. Nanofibers deposited on the collecting substrate can be transferred to working substrates using various transfer methods, such as contact printing (which is described in the next chapters). Once transferred, the nanofibers can be patterned and utilized in the fabrication of devices.

In the pursuit of electronic devices with low sheet resistance, long continuous paths, and low threshold voltage, researchers have explored electrospinning. For instance, Liu et al. fabricated several hundred micrometer‐long lithium lanthanum titanate (Li_0.33_La_0.557_TiO_3_, LLTO) NWs and aligned them along an electrical path to improve the conductivity and reliability of electrolytes for the solid‐state lithium‐ion battery as shown in Figure 4a.^[^ ^46^ ^]^ In a different study, Hsu et al. combined electrospinning with electroless deposition to form an ultra‐long metal NW network (Figure 4b).^[^ ^47^ ^]^ Electrospun NWs were utilized either as they were or as seeding areas for electroless deposition. Electrospinning enabled the integration NWs on flexible and soft substrates, and additional electroless deposition helped reduce the sheet resistance. Moreover, electrospun NWs have been demonstrated as effective masking materials. Jeon et al. deposited aligned polymer NWs on a graphite sheet by applying a parallel electric field, as shown in Figure 4c.^[^ ^48^ ^]^ By carefully controlling the etching rate, they successfully achieved graphene NWs with a width of 63 nm, which was thinner than polymer NW used as masking materials. Another research by Hsu et al. focused on transparent electrodes.^[^ ^49^ ^]^ The researchers aimed to reduce the sheet resistance of the electrodes while maintaining optical transmittance (Figure 4d). They achieved both by combining spin‐coated AgNWs with widths ranging from 50 to 300 nm and electrospun mesoscale copper (Cu) NWs with widths ranging from 1 to 5 µm.

The capability of electrospinning for large‐scale fabrication, optimization of sheet resistance, and preservation of optical properties creates new ways for the design and manufacturing of advanced nanoelectronics.

### 2.2 Assembly of 1D Nanostructures by Contact Methods

Dispersion methods offer simplicity and the ability to fabricate nanodevices over large areas. However, they often require elaborate and complicated control of nanomaterial solutions. To address this challenge, several methods have been developed to enable more sophisticated integration, direct transfer, or manipulation of nanomaterials. These methods include contact printing, roll‐to‐roll printing, and atomic force microscopy (AFM).^[^ ^15^, ^49^, ^50^, ^51^, ^52^, ^53^, ^54^ ^]^

#### 2.2.1 Contact Printing

Contact printing is a mechanical transfer method for nanomaterials from a donor substrate to a target substrate. It is based on direct contact and typically involves shear force and normal pressure. Nanomaterials are transferred to the target substrate with strong adhesion and aligned along the direction of the external force. Contact printing is suitable for assembling multiple nanomaterials over a large area or a single nanomaterial in an extremely fine area. Therefore, it can be applied to fabricate various devices ranging from single NW sensors to NW circuits.

**Figure 5:**
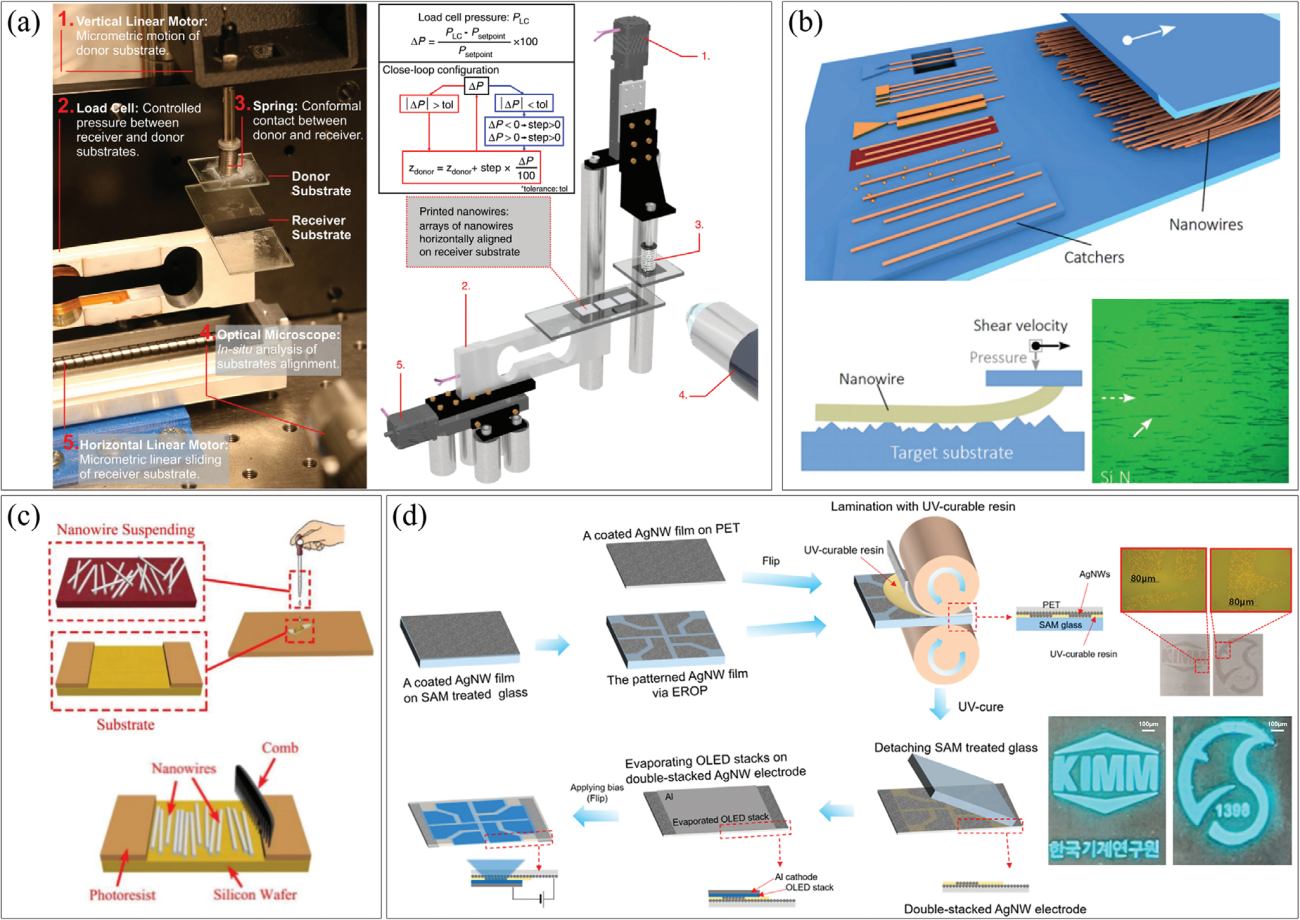
Assembly of 1D nanostructures by contact printing. a) (left) Image and (right) schematic illustration of the contact‐printing system. Reproduced with permission.^[^ ^50^ ^]^ Copyright 2018, The Authors, Published by Springer Nature Limited. b) Schematic illustration of (top) various catcher concepts and (middle) mechanical contact between a NW and target surface, and (bottom) optical microscopy image of NWs transferred onto a Si_3_N_4_ substrate. Reproduced with permission.^[^ ^15^ ^]^ Copyright 2016, IOP Publishing Ltd. c) Schematic illustration of (top) dropping of NW suspension on the substrate and (bottom) combing process for alignment. Reproduced under the terms of the CC‐BY license.^[^ ^55^ ^]^ Copyright 2018, The Authors, Published by MDPI. d) (left) Fabrication process of double‐stacked AgNWs layer device by sequential contact‐printing, and (right) optical images of the devices. Reproduced with permission.^[^ ^56^ ^]^ Copyright 2021, American Chemical Society.

Núñez et al. developed a precise contact printing system for controlling accurate positioning and force, as shown in **Figure** **5**a.^[^ ^49^ ^]^ They synthesized high‐quality and high‐aspect‐ratio zinc oxide (ZnO) NWs and SiNWs using bottom‐up and top‐down approaches, respectively, and transferred them to the target substrate. The NWs could be transferred to both rigid and flexible substrates and were utilized as UV photodetectors. Roßkopf et al. developed a surface‐controlled contact printing technique based solely on the friction between individual NWs on the donor substrate and the target substrate, as depicted in Figure 5b.^[^ ^15^ ^]^ When the frictional force exceeded a certain threshold, the NWs detached from the donor substrate and transferred to the target surface. Local friction force was controlled by surface features called catchers, obtained through mask‐assisted photolithography. The catchers influenced the local load or tribological contact morphology depending on their material composition and height, resulting in the positioning of each individual NWs. This method allowed for printing nanomaterials in the desired pattern over a large area. Cheng et al. introduced a simple method to align NWs without requiring precise equipment.^[^ ^55^ ^]^ They dispersed ZnO NWs on a substrate with grooves and swept them with a makeup brush. Some NWs were trapped inside the grooves and forced to assemble in an orderly manner, while the remaining NWs were swiped away from the grooves, as shown in Figure 5c. This combing process enabled over 80% of the NWs to be aligned within 20° angles for the fabrication of FET devices. Sequential contact printing can be utilized for the fabrication of more sophisticated devices. Kim et al. demonstrated the fabrication of devices consisting of double‐stacked AgNWs layers using contact printing twice.^[^ ^56^ ^]^ A resin used in the second printing step supported the multi‐layer structure, as illustrated in Figure 5d. This structure was employed as transparent electrodes for organic light‐emitting diodes (OLEDs) with various patterns.

#### 2.2.2 Roll‐to‐Roll Printing

Roll‐to‐roll printing is a technique similar to contact printing, but it utilizes a roll‐formed substrate, such as a flexible plastic or metal foil, allowing for the fabrication of long patterns over a continuous length.

**Figure 6:**
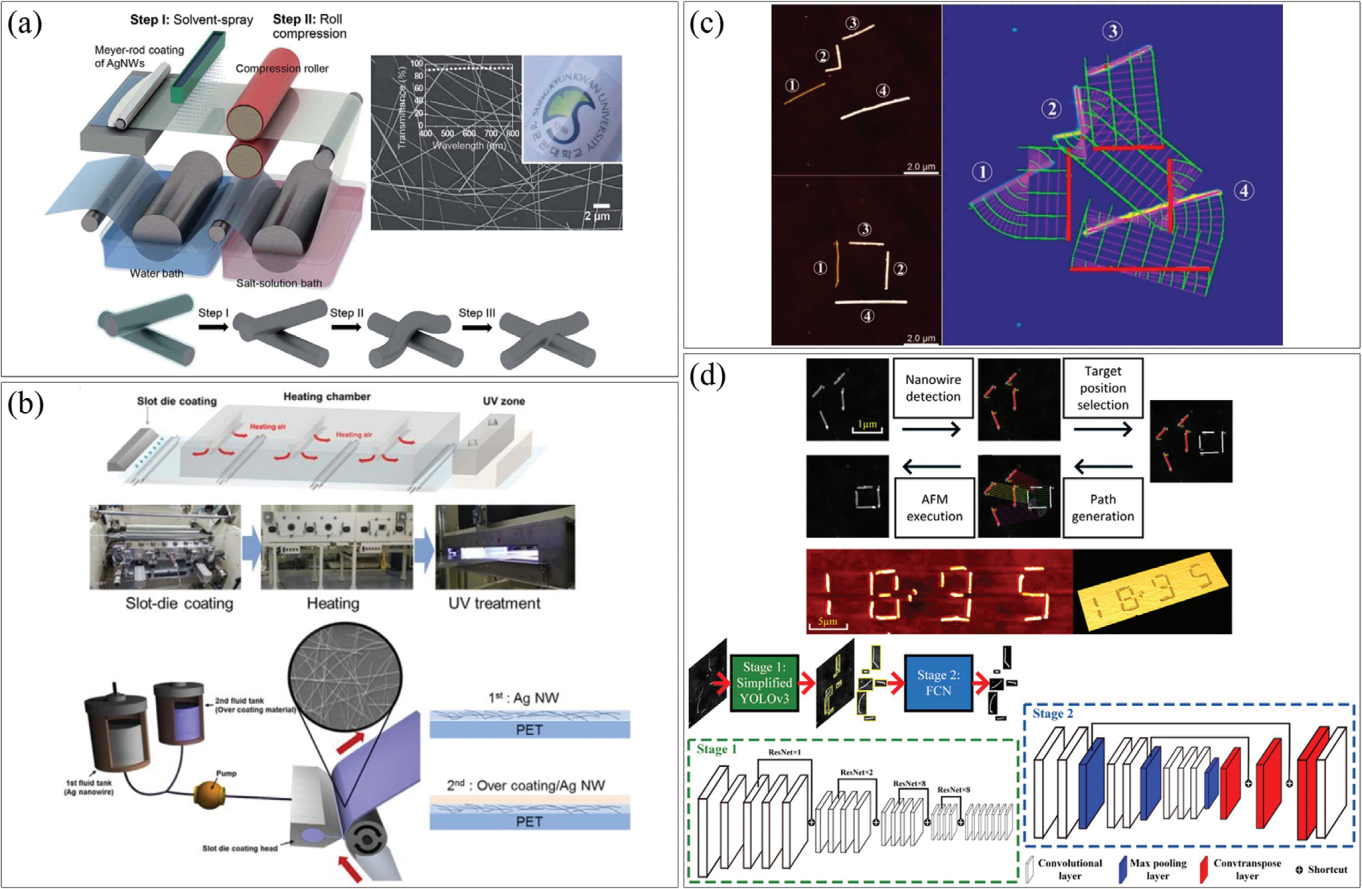
Assembly of 1D nanostructures by roll‐to‐roll printing. a) (top left) Schematic illustration of the roll‐to‐roll welding process for AgNW electrodes, (top right) SEM image of the AgNWs electrode, and (bottom) schematic illustration of AgNW welding. Reproduced with permission.^[^ ^52^ ^]^ Copyright 2014, Royal Society of Chemistry. b) Schematic illustration and photo of (top) the continuous roll‐to‐roll system and (bottom) slot‐die coating of AgNW and over‐coating layer. Reproduced with permission.^[^ ^53^ ^]^ Copyright 2016, Springer Nature Limited. Assembly of 1D nanostructures using AFM. c) (left) Photos of AgNWs before and after manipulation and (right) the automated image processing for manipulating desired positions of each NW based on parallel pushing vectors using MATLAB. Reproduced with permission.^[^ ^54^ ^]^ Copyright 2017, IOP Publishing Ltd. d) (top) Demonstration of the automated nanomanipulation technique and (bottom) architecture of the segmentation network consisting of YOLOv3 and FCN. Reproduced with permission.^[^ ^58^ ^]^ Copyright 2021, AIP Publishing.

Meng et al. successfully fabricated a 5‐m‐long nanomaterial‐patterned device, resulting in a significant reduction in fabrication cost.^[^ ^57^ ^]^ Roll‐to‐roll printings have been widely employed for the fabrication of electrodes and functional films.^[^ ^51^, ^52^, ^53^, ^57^ ^]^ Nanomaterials suitable for roll‐to‐roll printing have been developed and have demonstrated satisfactory performance despite the large area printing and low production cost. A notable example is the use of AgNWs as a replacement for indium tin oxide (ITO) due to their flexibility, which is well‐suited for the roll‐to‐roll process. Lee et al. fabricated AgNW network films using a roll‐to‐roll process consisting of three steps: solvent injection, roll compression, and salt treatment/washing.^[^ ^52^ ^]^ Compared to ITO, AgNWs formed a percolating network with low density and exhibited high conductivity. The salt‐treatment process enhanced the contact strength between AgNWs (**Figure** **6**a). Since roll‐to‐roll printing is a continuous process, it can be easily combined with other pre‐ or post‐treatment processes. Kim et al. fabricated a large‐area touch screen panel using a roll‐to‐roll slot die coater.^[^ ^53^ ^]^ AgNWs were coated on the substrate for electrodes, and an over‐coating layer was sequentially applied to enhance durability against erosion, as shown in Figure 6b.

#### 2.2.3 Atomic Force Microscopy‐Based Manipulation

Another method within the category of contact printing is AFM‐based manipulation. AFM is a powerful tool known for its ability to observe and manipulate nanoscale objects with sub‐nanometer resolution.

Liu et al. conducted manipulation of NWs by analyzing real‐time data obtained from AFM and moving the NWs in an optimized sequence (Figure 6c).^[^ ^54^ ^]^ Image processing techniques, including edge detection, filling, and skeleton extraction, were utilized to identify the NWs from the AFM images. The identified NWs were then manipulated using a series of short parallel pushing vectors, enabling precise translation and rotation to position individual NWs at desired locations. In this process, the next point at which to apply a force is determined by observing the movement of the NW. Precise movement controls usually require complex and intensive calculations. To address this, Bai et al. applied deep learning techniques to detect the posture and position of NWs.^[^ ^58^ ^]^ They utilized a real‐time object detection algorithm known as simplified You Only Look Once version 3 (YOLOv3) to predict the shape of NWs and obtain their bounding boxes. Additionally, a tiny fully convolutional network (FCN) was employed to generate object masks (Figure 6d).

The assembly of 1D nanostructures through contact methods provides a versatile approach for nanodevice fabrication. However, it is important to acknowledge the limitations of contact methods. Some of these techniques require precise alignment and control of nanomaterials, which can be both challenging and time‐consuming. The application of shear force and pressure during contact printing may pose risks of potential damage to the nanomaterials. Additionally, complex patterns or intricate designs may not be easily achievable using these methods. In the upcoming chapter, we explore non‐contact methods for nanomaterial assembly, offering alternative approaches to overcome some of these limitations.

### 2.3 Assembly of 1D Nanostructures by Microfluidic Forces

Traditionally, one of the most popular techniques for integrating NWs is through the use of microfluidics and fluid drag forces. In this method, shear forces align the NWs as the solution flows through microfluidic channels. Recent research has expanded the scope of microfluidic techniques, particularly by leveraging capillary force. This force plays a crucial role in guiding the alignment process during solution drying. Furthermore, these microfluidic approaches are often combined with surface treatment and/or external fields in configurations that synergistically enhance alignment yield, enable larger‐scale fabrication, and accelerate the assembly processes.

#### 2.3.1 Fluid Drag Forces

**Figure 7:**
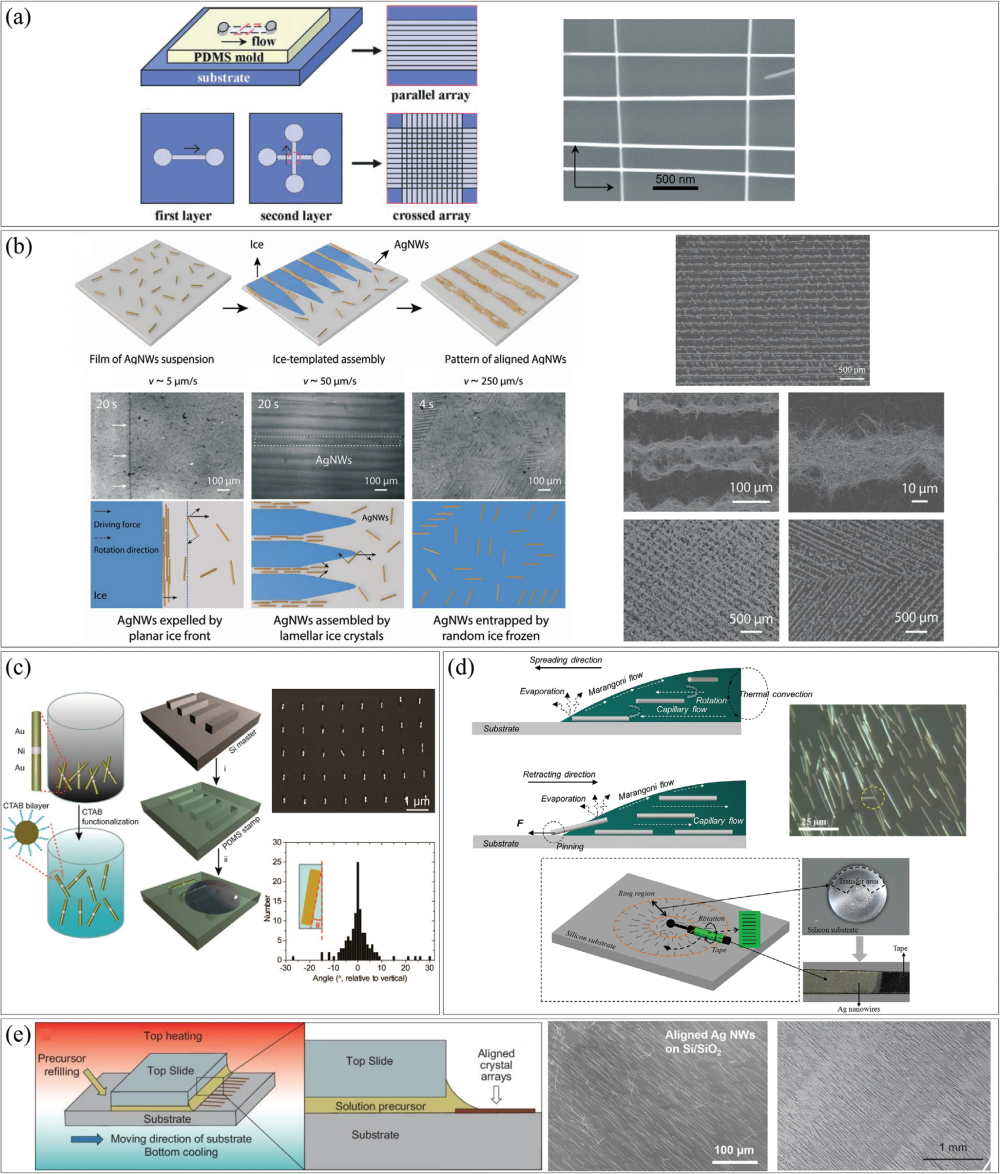
Assembly of 1D nanostructures by fluid drag forces. a) Schematic illustration of (left top) PDMS mold with flow channels for flow‐assisted assembly and (left bottom) sequence of two‐step sequential flow assisted assembly for crossed arrays, and (right) SEM images of an orthogonally crossed array of n‐type InP NWs using flow‐assisted assembly. Reproduced with permission.^[^ ^59^ ^]^ Copyright 2001, The American Association for the Advancement of Science. b) (top left) Schematic illustration of the assembly process of AgNWs by lamellar freezing, (bottom left) in‐situ optical observation and mechanism analysis of the ice‐templated assembly of AgNWs depending on ice growth velocity, and (right) SEM images of fabricated AgNWs electrodes. Reproduced with permission.^[^ ^62^ ^]^ Copyright 2021, Wiley‐VCH GmbH. Assembly of 1D nanostructures by capillary forces. c) (left) Schematic illustration of the alignment of multi‐segmented NWs into trenches by capillary forces, (right top) SEM image of aligned NW array consisting of two Au segments separated by a short Ni segment and (right bottom) distribution of the orientation of aligned NWs. Reproduced with permission.^[^ ^63^ ^]^ Copyright 2014, American Chemical Society. d) (top left) Schematic illustration of AgNWs align process by evaporation and (top right) SEM image of aligned NWs and (bottom) schematic illustration and the result of NW transfer process. Reproduced with permission.^[^ ^64^ ^]^ Copyright 2014, The Authors, Published by Springer Nature Limited. e) (left) Schematic illustration of AgNWs align process by moving stage and top–heating–bottom‐cooling (THBC) conditions, SEM image of (middle) aligned AgNWs and (right) directionally synthesized CH_3_NH_3_PbI_3_ NWs. Reproduced with permission.^[^ ^65^ ^]^ Copyright 2019, Oxford University Press on behalf of China Science Publishing & Media Ltd.

The potential of fluid drag forces in controlling the alignment of NWs was first demonstrated by Huang et al.^[^ ^59^ ^]^ In their study, the authors successfully controlled the separation between NWs (gallium phosphide (GaP), indium phosphide (InP), and SiNWs) to as low as 400 nm with an angular spread of less than 6°. **Figure** **7**a illustrates the PDMS molds utilized to guide a solution containing the 1D nanomaterials, with the NWs aligning in the direction of the drag forces they experienced. They also showed the formation of orthogonally‐crossed arrays of n‐type InP NWs through a sequential two‐step flow, while other angles could be achieved by rotating the PDMS mold. The degree of alignment and NW density could be controlled by adjusting the flow speed and duration, respectively. In order to achieve this result, the surface of all target substrates in the work was functionalized with an NH_2_‐terminated self‐assembled monolayer (SAM). Subsequent to Huang et al.’s work, numerous researchers have employed fluid drag forces to align 1D nanomaterials.^[^ ^60^, ^61^ ^]^ Practical devices, including semiconducting resistive NWs and diodes by bringing p‐ and n‐junctions in contact, have been successfully constructed using similar techniques. The main advantage of using microchannels (and employing drag forces) is their simplicity and cost‐effectiveness, as no prefabricated electrodes are required (unlike the dielectrophoresis method, which is discussed later). However, it is important to note that the width of the microchannel in the PDMS mold sets a limitation on the minimum attainable patterns. Han et al. introduced a unique method for creating a directional flow that aligns NWs (Figure 7b).^[^ ^62^ ^]^ They dispersed an aqueous suspension of SiNWs on a polyethylene terephthalate (PET) film, with one end of the substrate being cooled. As the water froze from that end, a controlled freezing speed of 40–60 µm s^−1^ was maintained to generate a lamellar ice pattern. If the freezing speed was slower or faster, it resulted in the generation of planar or random structure ice, respectively. When the freezing speed was moderate, patterned ice perpendicular to the cooling direction was formed, and during this freezing process, the AgNWs were pushed by the solution and aligned in parallel to each other. The authors utilized these aligned NWs as electrodes for flexible devices.

#### 2.3.2 Capillary Forces

Capillary forces arise at the interface between liquid‐to‐liquid and liquid‐to‐solid, and they become stronger as the size of the object decreases. Zhou et al. fabricated PDMS trenches with lengths ranging from 300 to 450 nm and widths of 70–80 nm.^[^ ^63^ ^]^ They then arranged individual Au‐Ni NWs, each with lengths ranging from 120 to 250 nm, within each trench (Figure 7c). Capillary‐force assembly is suitable for large areas, but it is governed by diffusion‐limited processes, which can take several hours to assemble a few inches as the NW suspension dries. Dai et al. introduced a simple method for aligning NWs based on the capillary effect and the Marangoni effect.^[^ ^64^ ^]^ They placed a solution of 1 wt% AgNWs in ethanol on a Si substrate and allowed it to evaporate naturally. As shown in Figure 7d, capillary and Marangoni forces acted on the NWs, aligning them radially with a standard deviation of 5.15°. The aligned NWs were harvested using equipment that combined a connecting rod and a rubber roller. This process was carried out at room temperature and normal pressure, and the alignment quality was controlled by the volume of the solution. Li et al. reported a method for fabricating substrates with directionally aligned NWs using the same principle (Figure 7e).^[^ ^65^ ^]^ A solution containing AgNWs in dimethylformamide (DMF) was passed through a narrow gap of 50 or 100 µm between an upper plate and a moving lower substrate. The drag force generated by the aligned flow from the moving substrate combed the NWs, and the NWs were fixed to the substrate by capillary force resulting from the evaporation of the solution. The upper plate was heated, and the bottom substrate was cooled to create a steady Marangoni flow with a single vortex, and the solution was continuously supplied for large‐scale fabrication. This method not only aligns pre‐synthesized NWs but also synthesizes aligned NWs by supplying a precursor solution. As the precursor solutions passed through the gap, nanomaterials were synthesized and their morphology, crystallinity, and density were determined by the concentration of the solution, the distance between the plates, and the temperature of the upper and lower substrates. Using this method, arrays of Methylammonium lead iodide (CH_3_NH_3_PbI_3_), 2,4,5‐triphenylimidazole (TPI), 9,10‐bis(phenylethynyl)anthracene (BPEA), and C_60_ crystals were fabricated, and their alignment was influenced by temperature, heat flux, and the type of solution.

Microfluidic force‐based approaches utilizing fluid drag forces and capillary forces have provided valuable methods for aligning NWs in device fabrication. These techniques offer notable advantages, including simplicity, cost‐effectiveness, and the ability to achieve large‐scale fabrication. However, it is important to consider their limitations. Microchannel‐based methods face constraints on the minimum attainable patterns dictated by the channel width, while capillary force assembly can be time‐consuming due to diffusion‐limited processes, resulting in longer assembly times. In addition, fluids by their nature have no fixed shape and the forces act on the surface of the material rather than on the material itself, which is detrimental to precise manipulation. Despite these drawbacks, microfluidic force‐based assembly methods remain a viable approach for the bulk assembly of a wide variety of nanomaterials. In addition, techniques that combine hydrodynamics with other forces to achieve sophisticated manipulation are being developed and will be introduced in subsequent sections. In the next chapter, we explore alternative methods that involve the direct application of electromagnetic forces to nanostructures for alignment purposes.

### 2.4 Assembly of 1D Nanostructures by Electromagnetic Forces

Electromagnetic fields offer a highly versatile integration method for nanomaterials in a wide range of applications, such as transistors, gas sensors, flexible transparent films, and electrically conductive micro/nano patterns. In recent literature, approaches based on dielectrophoresis, magnetic fields, and electrohydrodynamic (EHD) printing have undergone extensive modifications to push the boundaries of these techniques.^[^ ^66^, ^67^, ^68^, ^69^, ^70^ ^]^ In this section, we will discuss how these modifications have enabled the achievement of higher performance and smaller devices, the creation of more complex patterns, improved control over device performance, increased throughput, and reduced manufacturing costs.

#### 2.4.1 Dielectrophoresis

Dielectrophoresis techniques make use of electric fields generated between two planar electrodes to align one or multiple 1D nanomaterials. These methods capitalize on the discrepancy in dielectric constants between the suspended nanomaterials and the surrounding solution, resulting in the generation of dielectrophoretic forces.

**Figure 8:**
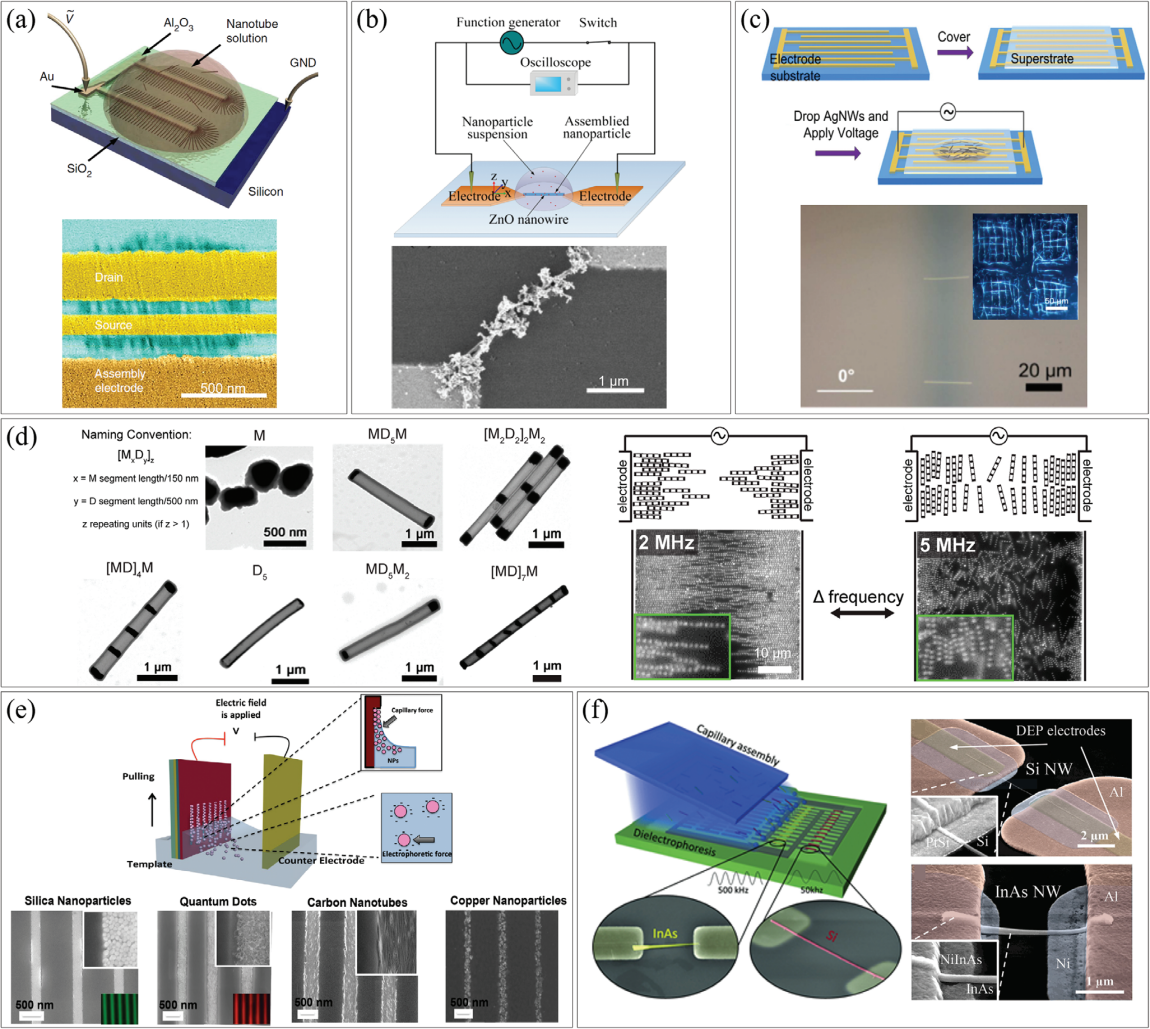
Assembly of 1D nanostructures by dielectrophoresis. a) (top) Schematic illustration of the fringing‐field dielectrophoretic assembly process and (bottom) SEM image of the constructed transistor. Reproduced with permission.^[^ ^66^ ^]^ Copyright 2014, Springer Nature Limited. b) (top) Schematic illustration of dielectrophoresis setup and (bottom) SEM image of the AuNP attached ZnO NW. Reproduced with permission.^[^ ^67^ ^]^ Copyright 2015, American Chemical Society. c) (top) Schematic illustration of the multi‐step dielectrophoresis process on a PET superstrate and (bottom) bright and dark field optical images of alignment of single NWs (first step) and NW chains in an orthogonal network configuration (second step). Reproduced with permission.^[^ ^68^ ^]^ Copyright 2017, IOP Publishing Ltd. d) (left) TEM images of various kinds of [MD]_x_M NWs and (right) schematic illustration and SEM images of aligned NWs in different orientations by applied AC frequencies. Reproduced with permission.^[^ ^71^ ^]^ Copyright 2020, American Chemical Society. Assembly of 1D nanostructures using dielectrophoresis combined with another force. e) (top) Schematic illustration of the electro‐fluidic assembly process and (bottom) SEM images showing line patterns of different materials achieved through electro‐fluidic assembly. Reproduced with permission.^[^ ^60^ ^]^ Copyright 2017, American Chemical Society. f) (left) Schematic illustration of the electro‐fluidic assembly process and (right) SEM images showing a SiNW and an InAs NW trapped between pairs of electrodes. Reproduced with permission.^[^ ^61^ ^]^ Copyright 2014, WILEY‐VCH Verlag GmbH & Co. KGaA, Weinheim.

Cao et al. achieved remarkable alignment results using fringing‐field dielectrophoretic assembly, with CNTs exhibiting an orientation within 6°, an average pitch of 21 nm, and a standard deviation of 6 nm.^[^ ^66^ ^]^ These exceptional alignment performances can be attributed to the self‐limited mechanism and the strong focus of the electric field. They aligned semiconducting CNTs using the fringing‐field dielectrophoresis method, as shown in **Figure** **8**a, which employs a different electrode configuration than conventional planar setups. Instead of using two planar metal comb‐type interdigitated electrodes, they utilized a top metal comb electrode and a Si substrate as the counter electrode. An intermediate thin thermal oxide dielectric layer (10 nm) was sandwiched between these electrodes. Additionally, the top electrode was coated with an 8 nm layer of aluminum oxide (Al_2_O_3_) to prevent electromigration. When a voltage was applied between the top and counter electrodes, the CNTs suspended in the solution aligned perpendicular to the surface of the top electrodes due to the strong fringing field generated. This fringing field was several times stronger than that in planar dielectrophoresis. In general, controlling the spacing of nanostructures using a dielectrophoresis method is challenging. However, they were able to achieve a 21 nm pitch in their devices because all the top electrodes were at the same voltage, and each aligned CNT screened the fringing field, preventing other CNTs from aligning in the same position. The authors utilized these aligned CNTs in FETs and demonstrated comparable device performance to those fabricated using complementary metal–oxide–semiconductor (CMOS) processes, with an *I*_on_/*I*_off_ ratio of 1000 and a peak transconductance of 52 µS µm^−1^. An alteration to conventional dielectrophoresis involves applying a two‐step process to coat NPs onto pre‐assembled 1D nanomaterials (Figure 8b). In this study, conducted by Ding et al., ZnO NWs were initially aligned across 3 µm gap electrodes, and subsequently, AuNPs were deposited on the surface of the aligned ZnO NWs by changing the suspension covering the electrodes and varying the excitation frequency.^[^ ^67^ ^]^ One advantage of this technique compared to others is the ability to control the number of particles deposited by manipulating the frequency of the alternating current (AC) signal during the second dielectrophoresis step. Using this approach, they developed UV detectors with a significantly improved *I*_on_/*I*_off_ ratio. For instance, when the atomic percentage of the coated NPs was approximately 40% (at a frequency of 500 kHz), the *I*_on_/*I*_off_ ratio increased from 10^2^ to 10^6^, and the rise and fall times of UV detection were also greatly enhanced. Trotsenko et al. developed a valuable approach in dielectrophoresis where a rigid and reusable substrate is employed to align nanostructure on a flexible superstrate.^[^ ^68^ ^]^ This method involved placing the flexible superstrate on top of the rigid substrate, enabling the alignment of nanostructure on different superstrates individually. Figure 8c illustrates the alignment of AgNWs on a flexible PET superstrate using dielectrophoresis forces induced by reusable electrodes on the underlying rigid substrate. This technique offered significant advantages for high‐throughput batch alignment of AgNWs on flexible substrates, particularly in applications such as transparent electrodes or transparent heaters. To ensure effective alignment, high frequencies (1 MHz) were employed to minimize the disruptive influence of electroosmotic flow, which might occur at lower frequencies. Additionally, ethanol was used and a constant AC excitation was maintained during the drying process to minimize the impact of capillary forces. Figure 8c shows both one‐step and multiple‐step alignments of NWs, corresponding to linear and network configurations, respectively. With the ordered network configuration, a 10% increase in transparency was achieved compared to randomly oriented NWs while maintaining low sheet resistances. The control over both the density and alignment direction of NWs can be achieved through innovative techniques. Famularo et al. synthesized NWs that combined metal and dielectric components using templated electrodeposition and successfully aligned them in various forms.^[^ ^71^ ^]^ The movement of NWs could be controlled by applying an AC signal, which interacted with the spacing between the metal and dielectric components of the NW. By adjusting the frequency of the AC signal, the alignment form of the NWs could be controlled. Figure 8d presents the results of this study. When a 2 MHz AC signal was applied to [MD]_4 _M NWs (where M represents metal and D represents dielectric), the NWs were aligned in parallel with the electric field direction. In contrast, when a 5 MHz AC signal was utilized, the NWs exhibited perpendicular alignment to the electric field direction.

By combining two or more forces, precise integration of nanomaterials into large areas can be achieved. One such method is electro‐fluidic directed assembly, as demonstrated by Yilmaz et al.^[^ ^60^ ^]^ In this approach, capillary forces were coupled with an electric field applied to plates, as depicted in Figure 8e, to assemble various nanomaterials (such as CNTs, silica NPs, cadmium selenide (CdSe) quantum dots, and CuNPs) on insulating substrates. As illustrated in the insets of Figure 8e, the micrometer‐scale PMMA pattern on the substrate drives the capillary force. Applying an electric field to the substrate increases assembly efficiency by tens of times. Controlling parameters such as electric field strength, solution pH and insulating layer thickness can increase assembly efficiency by 100 times compared to fluidic assembly alone. The authors demonstrated the application of CNTs for the detection of nitrogen dioxide (NO_2_) at concentrations as low as 1 ppm. Additionally, single‐line patterns could be achieved on transparent substrates, opening possibilities for flexible electronics applications. Notably, this technique eliminated the need for planar conductive electrodes commonly used in conventional dielectrophoresis, although an underlying conductive layer was required for bias application. Another modification of conventional microfluidic alignment is capillary‐assisted dielectrophoresis, as conducted by Collet et al.^[^ ^61^ ^]^ This technique combined dielectrophoresis and capillary forces to align NWs with pre‐fabricated electrodes. The study showed a five‐fold improvement in alignment yield on a large scale (over 500 NWs) compared to conventional dielectrophoresis. As shown in Figure 8f, during the process, a dummy substrate was moved on top of the target substrate, with a solution containing different nanomaterials in between. The NWs could be selectively aligned by applying specific frequencies between the electrodes. For example, in a suspension containing NWs of different materials, the electrodes could be programmed at different frequencies (e.g., 500 kHz for indium arsenide (InAs) and 50 kHz for Si) to align the desired NWs at specific electrode pairs. This technique offered the advantage of large‐scale integration with small feature sizes. Furthermore, the ability to integrate different nanomaterials by applying different frequencies enabled the construction of devices such as complementary transistors on a large scale.

#### 2.4.2 Magnetic Fields

An alternative method for aligning NWs is through the use of magnetic fields, offering simplicity, low cost, and high throughput. The magnetic force from permanent magnets can be applied to align magnetic NWs in a solution or a polymer matrix. Previous research in this field has primarily focused on the synthesis and alignment of magnetic nanomaterials, including ferromagnetic, paramagnetic, or diamagnetic materials.^[^ ^69^ ^]^

**Figure 9:**
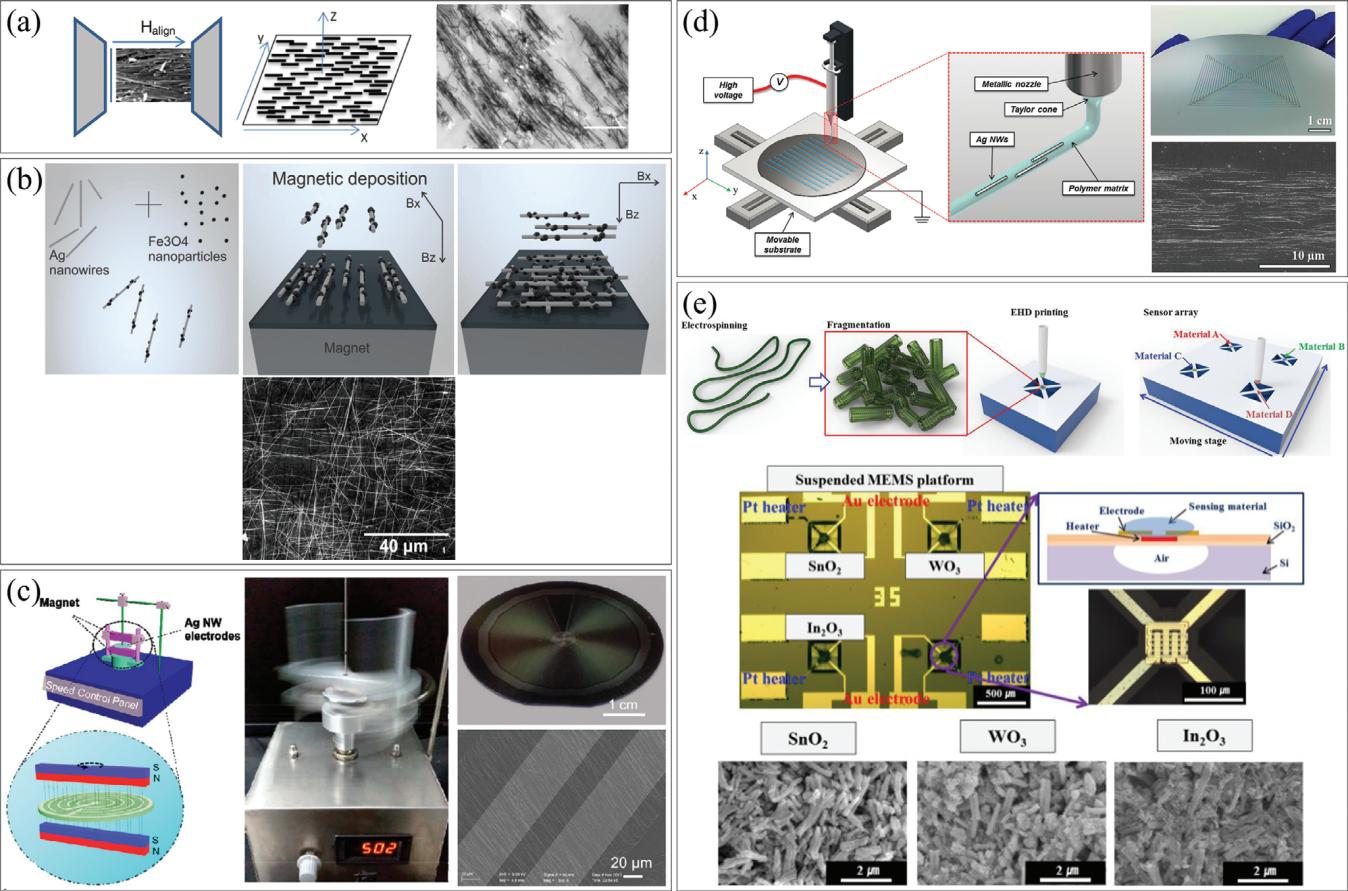
Assembly of 1D nanostructures by magnetic fields. a) (left) Schematic illustration of general magnetic field align process, and (right) SEM image of aligned Co NWs. Reproduced with permission.^[^ ^72^ ^]^ Copyright 2014, Springer Science Business Media Dordrecht. b) (top) Schematic illustration of magnetic assembly of Fe_3_O_4_ coated AgNWs, and (bottom) SEM image of two subsequent orthogonal alignments of NWs. Reproduced with permission.^[^ ^70^ ^]^ Copyright 2015, The Royal Society of Chemistry. c) (left) Schematic illustration and (middle) photo of electromagnetic induction generator (EMIG) device, and (right top) photo and (right bottom) SEM image of Ag NWs aligned in the direction of rotation. Reproduced with permission.^[^ ^74^ ^]^ Copyright 2020, American Chemical Society. Assembly of 1D nanostructures by electrohydrodynamic (EHD) printing. d) (left) Schematic illustration of the EHD system with movable stage, (right top) optical image of complex EHD printed pattern of AgNWs and (right bottom) their SEM image. Reproduced with permission.^[^ ^76^ ^]^ Copyright 2014, WILEY‐VCH Verlag GmbH & Co. KGaA, Weinheim. e) (top) Schematic illustration of the fabrication steps of multiple metal oxide fiber array through EHD printing and (middle) optical image of the suspended membrane with deposited nanomaterials (SnO_2_, WO_3_, and In_2_O_3_) and (bottom) their SEM images. Reproduced with permission.^[^ ^77^ ^]^ Copyright 2017, Elsevier.

Fang et al. introduced a general magnetic field‐induced alignment process using cobalt (Co) NWs.^[^ ^72^ ^]^ In their study, CoNWs of lengths ranging from 200 to 300 nm were mixed with polymers and placed between the polar pieces of an electromagnet. A magnetic field ranging from 0 to 2 T was applied, resulting in the alignment of CoNWs along the direction of the magnetic field, as shown in **Figure** **9**a. Notably, magnetic field‐based alignment eliminates the need for pre‐fabricated electrodes in the alignment process. More recent research has explored the functionalization of non‐magnetic 1D nanomaterials with ferromagnetic materials to achieve transparent films with unique electrical or magnetic properties.^[^ ^70^, ^73^ ^]^ In order for materials to align, it is necessary for the materials to response to the magnetic field. Ferromagnetic materials are particularly desirable for alignment purposes because they can derive strong magnetic forces. On the other hand, non‐magnetic materials require functionalization with magnetic materials prior to the alignment process. In one example, non‐ferromagnetic AgNWs were coated with ferromagnetic iron(III) oxide (Fe_3_O_4_)) NPs.^[^ ^62^ ^]^ The AgNWs were mixed with Fe_3_O_4_ NPs at various ratios (1:1 or 2:1) using polyethyleneimine (PEI) as a binding agent. Figure 9b shows the alignment of AgNWs coated with Fe_3_O_4_ NPs using permanent magnets with a magnetic field strength of 10.7 T m^−1^. The alignment process resulted in linear and mesh‐like patterns achieved through successive alignment in orthogonal directions. The NW mesh exhibited comparable sheet resistance and transmittance to those obtained through dielectrophoresis. However, in this study, an additional step was required to remove the introduced ferromagnetic materials. Fe_3_O_4_ NPs were removed using an oxalic acid solution. Afterward, the thermal annealing process at 150–200 °C for 20 to 30 min fused the NW junctions, enhancing the conductivity and mechanical robustness. In another approach, researchers have explored changing the direction of the magnetic field instead of using a fixed magnetic field. Wang et al. dispersed AgNWs in a suspended solution onto a substrate with patterned Au electrodes.^[^ ^74^ ^]^ The substrate was placed between two rotating permanent magnets positioned above and below it, rotating at ≈1000 rpm. The magnetic field attracted the AgNWs in the direction of rotation, resulting in their directional alignment, as depicted in Figure 9c.

#### 2.4.3 Electrohydrodynamic (EHD) Jet Printing

EHD jet printing operates similarly to the traditional inkjet printing method described above. In EHD jet printing, a high voltage is applied between the nozzle and the substrate, creating a drawing force that forms a thinner jet.^[^ ^75^ ^]^ It is a key difference compared to inkjet printing. As a result, it becomes possible to print lines narrower than the diameter of the nozzle, making this method widely used in nanodevice fabrication. Figure 9d illustrates the formation of arbitrary patterns using EHD jet printing with a movable stage.^[^ ^76^ ^]^ Typically, a voltage of 1–2 kV was applied between the needle and the substrate, with a gap of several hundred micrometers.^[^ ^75^, ^76^, ^77^ ^]^ To make the ink, a polymer matrix (polyethylene oxide, PEO) was mixed with AgNWs and (DI) deionized water. After the ink was jetted, the pattern was heated at 150 °C for 10 min, and the PEO was removed with ethanol to obtain aligned NWs. The alignment of the NWs was mainly a result of the shear forces they experienced in the flow of the EHD fiber jet. These forces compelled each AgNW to align parallel to the flow direction, minimizing hydrodynamic drag forces. Figure 9d demonstrates the successful fabrication of a two‐dimensional complex pattern with a line width in the tens of micrometers. The density of the NWs within the pattern could be controlled by the density of the NWs in the suspended solution. Linearly aligned AgNWs were obtained. A significant contribution of this work lies in its capability to pattern large‐area substrates with complex geometries. However, the low throughput of the EHD method may limit its practical large‐scale applications. Recently, EHD printing has also been used to deposit various metal oxide nanofibers, such as tin oxide (SnO_2_), tungsten oxide (WO_3_), and indium oxide (In_2_O_3_), in sub‐50 µm dot patterns onto micro‐electro‐mechanical systems (MEMS) platforms for gas sensing applications.^[^ ^77^ ^]^ Figure 9e illustrates the process, where metal oxide nanofibers were first synthesized via an electrospinning process, followed by a high‐temperature sintering process to decompose polymer additives. The nanofibers were then sonicated in ethanol to form short fragments, dried, and finally suspended in a solvent for EHD printing. Due to the high printing resolution of the EHD method, three kinds of materials could be separately integrated into a small area. The authors, Kang et al., first reported the successful patterning of metal oxide fibers using EHD printing and demonstrated the usefulness of an EHD‐printed metal oxide array on a microheater platform for a gas sensor array.

The disadvantages of the EHD method include cumbersome preparation processes and low throughput. Precise alignment of the jetting needle to the desired area and preparation of the nanomaterial ink for jetting is required. However, when combined with recent artificial intelligence (AI)‐based automated precision stage control technology, it is expected to overcome these challenges and become suitable for mass production. Additionally, since EHD can fabricate 3D structures, its usability can be increased by incorporating NWs.^[^ ^78^, ^79^ ^]^

### 2.5 Assembly of 1D Nanostructures by Optical Methods

When light is irradiated onto a nanomaterial, it undergoes refraction or reflection, resulting in the movement of the nanomaterial through its interaction with light. This phenomenon enables the manipulation of nanomaterials without the need for contact forces. The attractive or repulsive forces acting on the nanomaterials are determined by the difference in refractive indices between the nanomaterial and the surrounding medium.^[^ ^80^ ^]^ Optical methods, which are based on non‐contact optical forces and do not require electrodes, nozzles, tips, microchannels, or magnetic materials, offer an alternative approach when the previously discussed methods are not feasible. However, manipulating large objects becomes challenging due to large viscous drag, and the manipulation speed is relatively low, often taking several tens of seconds for an object.^[^ ^81^ ^]^ Several studies have achieved remarkable results using a technique known as “optical tweezers”, which will be further explained in the following section.

#### 2.5.1 Optical Tweezer

**Figure 10:**
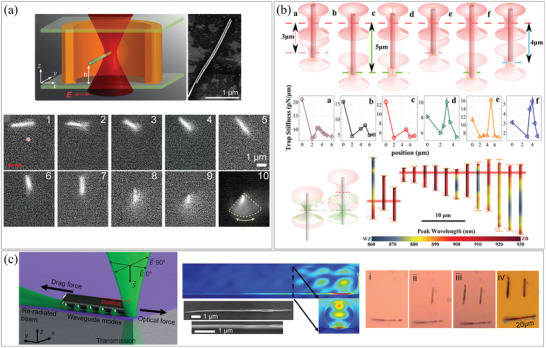
Assembly of 1D nanostructures using optical tweezer. a) (top left) Schematic illustration of optical trapping of AuNW by a single laser beam, (top right) SEM image of AuNWs manipulated by optical tweezer, and (bottom) the results of three‐dimensional optical trapping with time step measurement. Reproduced with permission.^[^ ^82^ ^]^ Copyright 2013, American Chemical Society. b) (top) Diagram of two spot trapping NWs and their axial trap stiffness measurement, and (bottom) axial NW photoluminescence mapping and trapping along z‐axis displacement. Reproduced with permission.^[^ ^83^ ^]^ Copyright 2013, American Chemical Society. c) (left) Schematic illustration of working principle of photonic nuzzling, (middle) electric field intensity and SEM image of NW and (right) sequential imaging of NW movement. Reproduced with permission.^[^ ^84^ ^]^ Copyright 2022, American Chemical Society.

Yan et al. demonstrated the manipulation of AuNWs with lengths exceeding 2 µm using a single‐beam optical tweezer with a wavelength of 800 nm (**Figure** **10**a).^[^ ^82^ ^]^ Previously, it was believed that large metal particles could not be trapped by optical tweezers due to their strong light absorption and scattering properties. Through their experiments and electrodynamics simulations, it was confirmed that the ability to trap metal nanostructures using optical tweezers depends on their plasmon properties. As a result, they successfully manipulated large nanostructures using this method. Wang et al. achieved stable control of long InP NWs with lengths ranging from 3 to 15 µm using trap multiplexing with two focused laser beams (Figure 10b).^[^ ^83^ ^]^ By employing a dynamic optical tweezer, they established a stable equilibrium trap position near the end of the InP NW based on the photoluminescent intensity along its length. Eliceiri et al. introduced a novel method called the photonic nozzling technique for manipulating NWs as shown in Figure 10c.^[^ ^84^ ^]^ They suspended SiNWs with diameters of 150–300 nm in DI water and dispersed them onto a thin Si‐coated quartz substrate. By maintaining a water thickness of ≈100 µm and irradiating a laser at the NW tip, the NWs acted as efficient waveguides, emitting light in the opposite direction of the laser irradiation and consequently moving in that direction. The authors also employed a nanosecond laser to achieve more effective movement while minimizing damage to the NWs.

### 2.6 Assembly of 1D Nanostructures by Substrate Deformation

In the previous chapters, we categorized the alignment methods for 1D nanostructures based on the type of force applied. However, in this chapter, we discuss alignment methods that are difficult to classify into a single category because they utilize multiple forces, primarily shear forces induced by substrate deformation.

#### 2.6.1 Langmuir–Blodgett Technique

**Figure 11:**
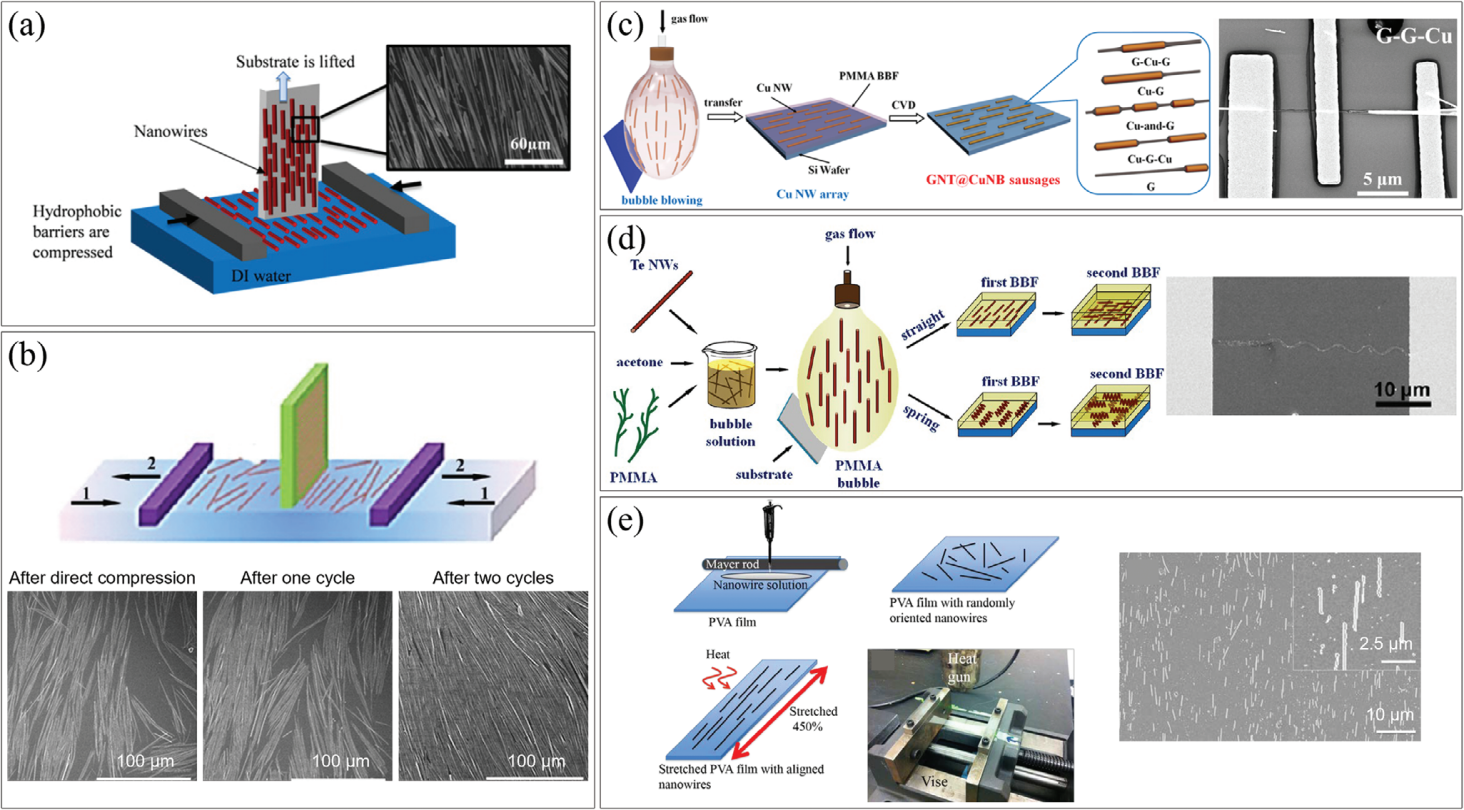
Assembly of 1D nanostructures using the Langmuir‐Blodgett technique. a) Schematic illustration of the LB setup for aligning GaN NWs, with inset SEM image showing the integrated GaN NWs monolayer. Reproduced with permission.^[^ ^85^ ^]^ Copyright 2014, IOP Publishing Ltd. b) (top) Schematic illustration of the compression‐expansion cycles for aligning the organic NTs and (bottom) SEM images of the aligned TMGE NTs after repeated compression‐expansion cycles. Reproduced with permission.^[^ ^86^ ^]^ Copyright 2016, American Chemical Society. Assembly of 1D nanostructures using blown bubble film (BBF) method. c) (left) Schematic illustration of the assembly process of CuNWs with BBF and their conversion to various graphene‐Cu nanostructures, and (right) SEM image of graphene‐graphene‐Cu NW device. Reproduced with permission.^[^ ^87^ ^]^ Copyright 2016, American Chemical Society. d) (left) Schematic illustration of the alignment process of TeNWs in linear and crossed configurations, and (right) SEM image of aligned TeNWs with a small angular deviation between samples. Reproduced with permission.^[^ ^88^ ^]^ Copyright 2014, American Chemical Society. e) (left) Schematic illustration of the alignment process using a stretching machine, and (right) SEM image of aligned AgNWs. Reproduced with permission.^[^ ^89^ ^]^ Copyright 2015, IOP Publishing Ltd.

The Langmuir–Blodgett (LB) method is particularly effective in creating highly dense arrays of well‐aligned NWs on specific surfaces. **Figure** **11**a illustrates the schematic of a vertical LB setup used for NW alignment.^[^ ^85^ ^]^ Salomon et al. utilized the piezoelectric properties of long gallium nitride (GaN) NWs to develop a flexible, self‐powered strain sensor. The device featured a capacitor‐like configuration, where the NWs were embedded in Parylene‐C (dielectric) and sandwiched between two electrodes. Initially, the NWs were grown via a CVD process, and their surfaces were modified to be hydrophobic before being suspended on the LB water surface. In the LB setup, polytetrafluoroethylene (PTFE) barriers were compressed, and the substrate was lifted to obtain a monolayer of NWs. One drawback of this technique is the potential for undesired agglomeration of NWs during the compression cycle. Organic NTs are generally more delicate and challenging to assemble compared to their inorganic counterparts. Zhou et al. introduced a sophisticated method for aligning various kinds of organic NTs.^[^ ^86^ ^]^ The working principle is similar to that of Salomon et al. but differs in that it uses repeated cycles of compression and expansion to obtain dense and well‐aligned nanostructures (Figure 11b). In this work, they use triethylene glycol monoethyl ether (TMGE) NTs as the basic structure. The TMGE NTs were prepared by a gelation process and were aligned using the LB process. To fabricate other types of aligned organic nanostructures, 2′,7′‐dichlorofluorescein sodium salt (DCFS), poly(N‐vinylcarbazole) (PVK) and perylene reagents were added to the TMGE NT gelation process. DCFS, PVK, and perylene were encapsulated in TMGE NTs and they were applied to the LB process for alignment. This showed that water soluble/oil soluble organic molecules can also be aligned by the LB process.

#### 2.6.2 Substrate Stretching

The blown bubble film (BBF) method has been utilized to align NWs within a polymeric film as the film undergoes expansion. Figure 11c depicts the basic steps of the BBF method. Initially, a polymer solution (in this case, PMMA) was mixed with pre‐synthesized NWs (in this case, Cu), and the film was then blown by introducing gas (in this case, air).^[^ ^87^ ^]^ The NWs were aligned in the direction of film expansion due to the shear forces experienced during the process. Subsequently, when the bubble came into contact with the target substrate (in this case, Si), the aligned nanomaterials could be transferred. In the subsequent CVD process, the PMMA film served as a source material and was converted into graphene nanotubes (GNTs), resulting in the formation of sausage‐like GNT@CuNB nanostructures. These structures hold potential for applications in large‐scale heterojunction electronic devices and high‐stability transparent electrodes. Wu et al. also developed a similar method using a PMMA film for the alignment of tellurium (Te) NWs, as shown in Figure 11d.^[^ ^88^ ^]^ However, in this case, the PMMA film was removed after the NWs were transferred to the target substrate via the BBF method. The scalability of the BBF method was demonstrated by the formation of large bubbles with diameters of 10–20 cm. With this approach, the NWs could be densely packed (average distance of 3.55 µm ± 1.4 µm) with a small angular deviation below 7°. Dong et al. achieved NW alignment using the BBF method with improved controllability (Figure 11e).^[^ ^89^ ^]^ They deposited AgNWs suspended in an ethanol solution onto a polyvinyl alcohol (PVA) film, which was then stretched to 450% of its original length. During the stretching process, the substrate was heated to 140 °C to facilitate stretching, and once the stretching was completed, the PVA film was maintained in its stretched state after the heat was removed. The NWs on the film were aligned in the tensile direction due to shear forces from the substrate and remained aligned even after the tensile force was removed. The aligned AgNWs were subsequently transferred to a PET film via a hot‐rolling process, and the orientational order parameter,^[^ ^90^ ^]^ which is defined as 0 for random alignment and 1 for perfect alignment, was measured to be 0.97.^[^ ^89^ ^]^

In general, the key advantages of substrate stretching techniques are their versatility in integrating materials and their ability to facilitate the large‐scale integration. On the other hand, the biggest drawback of this method is that it is the inherent challenge of achieving precise alignment of NWs at specific desired locations.

## 3 Direct Integration of 1D Nanostructures

Pre‐synthesis and post‐assembly methods explained above continue to be widely used for NW‐based device fabrication. These methods can accommodate most types of NWs since they utilize pre‐synthesized NWs, allowing for straightforward functionalization through doping and coating procedures. However, these approaches have limitations due to the complex and low‐throughput assembly process. Achieving both large‐area integration and precise assembly simultaneously is indeed challenging, and the assembly methods are constrained by the properties of the nanomaterials. Additionally, the bonding between nanomaterials and substrates relies primarily on weak van der Waals forces, making it difficult to establish robust mechanical and electrical connections. To overcome these drawbacks, researchers have devised direct synthesis and integration methods and conducted studies on the electrical reliability and mechanical robustness of the integrated region.^[^ ^91^ ^]^ Two techniques are employed for direct synthesis and integration: one involves pre‐designating the area for nanostructure synthesis using patterned seed or catalyst layers, and the other entails creating local hotspots to induce nanomaterial synthesis.

### 3.1 Integration of NWs by Patterning

Most nanomaterials can be synthesized by epitaxial growth from liquid or gaseous precursors, generating their own nuclei as a starting point for their growth. To synthesize nanomaterials in specific areas, it is necessary to designate the nucleation point before synthesis. This can be achieved by depositing a seed or catalyst material, or by physically marking or damaging the substrate to create an unstable region that serves as a nucleation point.

#### 3.1.1 Seed/Catalyst Patterning and Selective Synthesis

**Figure 12:**
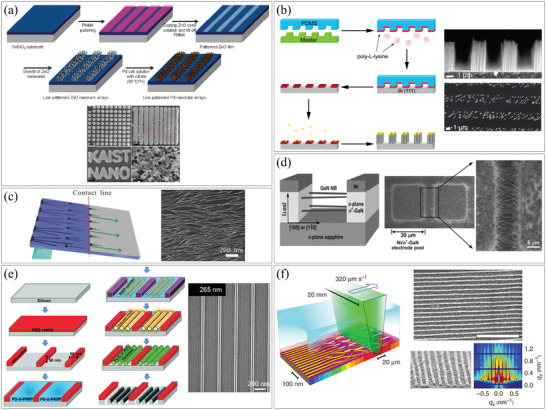
Direct synthesis and integration of 1D nanostructures by seed patterning and synthesis. a) (top) Fabrication procedure of Pd NT pattern designated by ZnO seed layer, and (bottom) SEM images of spot, line and letter patterns. Reproduced with permission.^[^ ^93^ ^]^ Copyright 2012, American Chemical Society. b) (left) Schematic illustration of the fabrication process of patterned Au colloids and SiNWs using a PDMS stamp, and (right) SEM images of SiNW array fabricated by patterned growth. Reproduced with permission.^[^ ^94^ ^]^ Copyright 2005, American Chemical Society. c) (left) Schematic illustration of aligned OIP NW growth by the evaporation‐induced self‐assembly method (EISA), and (right) SEM image of horizontally aligned OIP NWs. Reproduced with permission.^[^ ^95^ ^]^ Copyright 2015, Royal Society of Chemistry. d) (left) Schematic illustration of the GaN nanobridge device, and (right) SEM images of GaN nanobridge connecting two functional posts. Reproduced with permission.^[^ ^96^ ^]^ Copyright 2008, Wiley‐VCH Verlag GmbH & Co. KGaA, Weinheim. Direct synthesis and integration of 1D nanostructures using block copolymer. e) (left) Schematic illustration of the nanopatterning fabrication process using block copolymer, and (right) SEM image of γ‐Al_2_O_3_ NWs. Reproduced with permission.^[^ ^97^ ^]^ Copyright 2015, Royal Society of Chemistry. f) (left) Schematic illustration of soft‐shear laser zone annealing (SS‐LZA) for NW fabrication using monolithically aligned block copolymer templates, and (right) SEM image of Pt NWs converted from P2VP cylinder. Reproduced with permission.^[^ ^98^ ^]^ Copyright 2015, Springer Nature Limited.

Patterning seeds or catalysts enables precise localization of NW synthesis. In the field of microfabrication processes, numerous methods have been developed for pre‐patterning and deposition. Referring to these processes, UV and E‐beam lithography are commonly used for seed and catalyst patterning in NW synthesis.^[^ ^92^ ^]^ Lim et al. introduced a patterning method for ZnO NPs using the lift‐off technique (**Figure** **12**a).^[^ ^93^ ^]^ They patterned a dual layer of positive PR/PMMA through UV photolithography and oxygen (O_2_) plasma etching. The ZnO nanocrystal seed layer was drop‐cast onto the patterned substrate several times, followed by the lift‐off process. Subsequently, ZnO NWs were selectively grown on the ZnO NP seed patterns via hydrothermal synthesis. Through a liquid phase deposition (LPD) process, the ZnO NWs were converted into palladium (Pd) NTs. Hochbaum et al. reported a patterning method using an elastomeric stamp (Figure 12b).^[^ ^94^ ^]^ They made a PDMS stamp with microscale line patterns (2 µm width line and 2 µm gap). A thin polyelectrolyte layer (poly‐L‐lysine) was transferred to Si substrates using the stamping method. The patterned substrate was immersed in a 50 nm Au colloidal solution for a short period, allowing the colloids to adhere electrostatically to the micropatterned areas of the polyelectrolyte. Finally, SiNWs were synthesized on this substrate through a CVD process. This method offers the advantage of being a straightforward contact printing approach without the need for repetitive photolithography processes.

However, these direct integration methods have limitations in certain applications because NWs typically grow vertically from the substrate. Deng et al. proposed a synthesis method for horizontally growing NWs on a substrate, along with their alignment in the desired direction through directional solvent evaporation (Figure 12c).^[^ ^95^ ^]^ To designate the synthesis area, the substrate was selectively treated with UV‐ozone to create a hydrophilic surface. The seed solution adhered to the treated area, and organolead iodide perovskite (OIP) NWs were synthesized on the seeded area using a solvothermal reaction. The NWs self‐aligned due to the directional evaporation of solvents, achieving precise positioning and desired alignment. Furthermore, Chen et al. synthesized NWs in the lateral direction by leveraging the epitaxial growth based on the crystalline plane of GaN (Figure 12d).^[^ ^96^ ^]^ Using photolithography, lift‐off, and reactive‐ion etching (RIE) processes, arrays of micro‐sized Ni/n^+^‐GaN electrode posts were patterned on an insulating sapphire substrate. The n^+^‐GaN posts exposed specific sidewalls with selected orientations, such as the a‐ and m‐facets. Subsequently, after Au catalyst sputtering, NWs were grown via a CVD process using ammonia (NH_3_) and gallium (Ga) as source reagents. The grown NWs extended between the facing sidewalls, forming bridges. This direct lateral growth method demonstrated excellent alignment and contact properties.

#### 3.1.2 Block Copolymer

In addition to lithography methods, nano‐sized patterns can be made using block copolymers. Block copolymers consist of two or more homopolymers linked by covalent bonds, forming repeating patterns. The separation of each polymer in the mixture occurs at a specific temperature and pressure, and the size and shape of the patterns are determined by the length of the polymer segments and the ratio of the mixture. Initially, polymers were predominantly used as they are, but metal or metal oxide patterns can also be fabricated using substitution and implantation methods.

Figure 12e illustrates the work of Cummins et al., who fabricated Fe_3_O_4_ NWs and Al_2_O_3_ NWs with widths of less than 10 nm through directional self‐assembly, surface reconstruction, and UV/O_3_ treatment.^[^ ^97^ ^]^ They annealed a toluene‐tetrahydrofuran (THF)‐based poly(styrene)‐block‐poly(4‐vinyl‐pyridine) (PS‐b‐P4VP) copolymer under chloroform (CHCl3) vapor, resulting in the formation of P4VP cylinders. Subsequently, the P4VP block copolymer was converted into Fe_3_O_4_ NWs and aluminum oxide (Al_2_O_3_) NWs using UV/ozone (O_3_) treatment. Majewski et al. proposed a method for fabricating Pt NWs using block copolymers and focused laser (Figure 12f).^[^ ^98^ ^]^ They prepared a polystyrene‐block‐poly(2‐vinyl pyridine) (PS‐b‐P2VP) copolymer on a germanium (Ge) coated substrate. A solid‐state green (532 nm) laser with a power of 3 W and a narrow line‐shaped zone was exposed to the substrate resulting in a thermal zone temperature estimated at 270 °C. This thermal energy‐activated phase separation and PS‐b‐P2VP cylindrical patterns were fabricated. The patterns were then converted into metal NWs through metal salt complexation, finally resulting in the synthesis of a Pt NWs array with a diameter of 12 nm.

These methods utilizing block copolymers allow for the easy fabrication of very long and thin NWs. However, they require pre‐processes to make guides for assisting the block copolymer patterning. Furthermore, the synthesized area is typically limited to tens of µm^2^, which poses challenges for practical applications.

### 3.2 Integration of NWs by Local Heating

Most of the methods mentioned in Section 3.1 apply heat to the entire device area and activate the synthesis reaction only in pre‐selected zones through patterning. As a result, pre‐ or post‐processes involving patterning are required, and undesired areas may be exposed to heat. In this section, we explore methods that enable the selective synthesis of NWs by focusing heat on specific areas. There are three mechanisms for transferring heat energy, but convection is not ideal due to the challenges of directional heat transfer. On the other hand, direct conduction of heat generated by Joule heating and focused radiation by laser is suitable for achieving local heating.

#### 3.2.1 Joule Heating

Several studies have reported on the precise integration and positioning of NWs in desired locations through localized thermal energy fields generated by pre‐integrated micro/nano heaters on a device. By applying an electrical voltage across the micro/nano‐sized heaters, localized temperature rise through Joule heating induces the reaction of precursor molecules surrounding the heaters. This approach allows for the efficient utilization of chemicals and energy in NW synthesis and requires a relatively simple and inexpensive fabrication setup.

**Figure 13:**
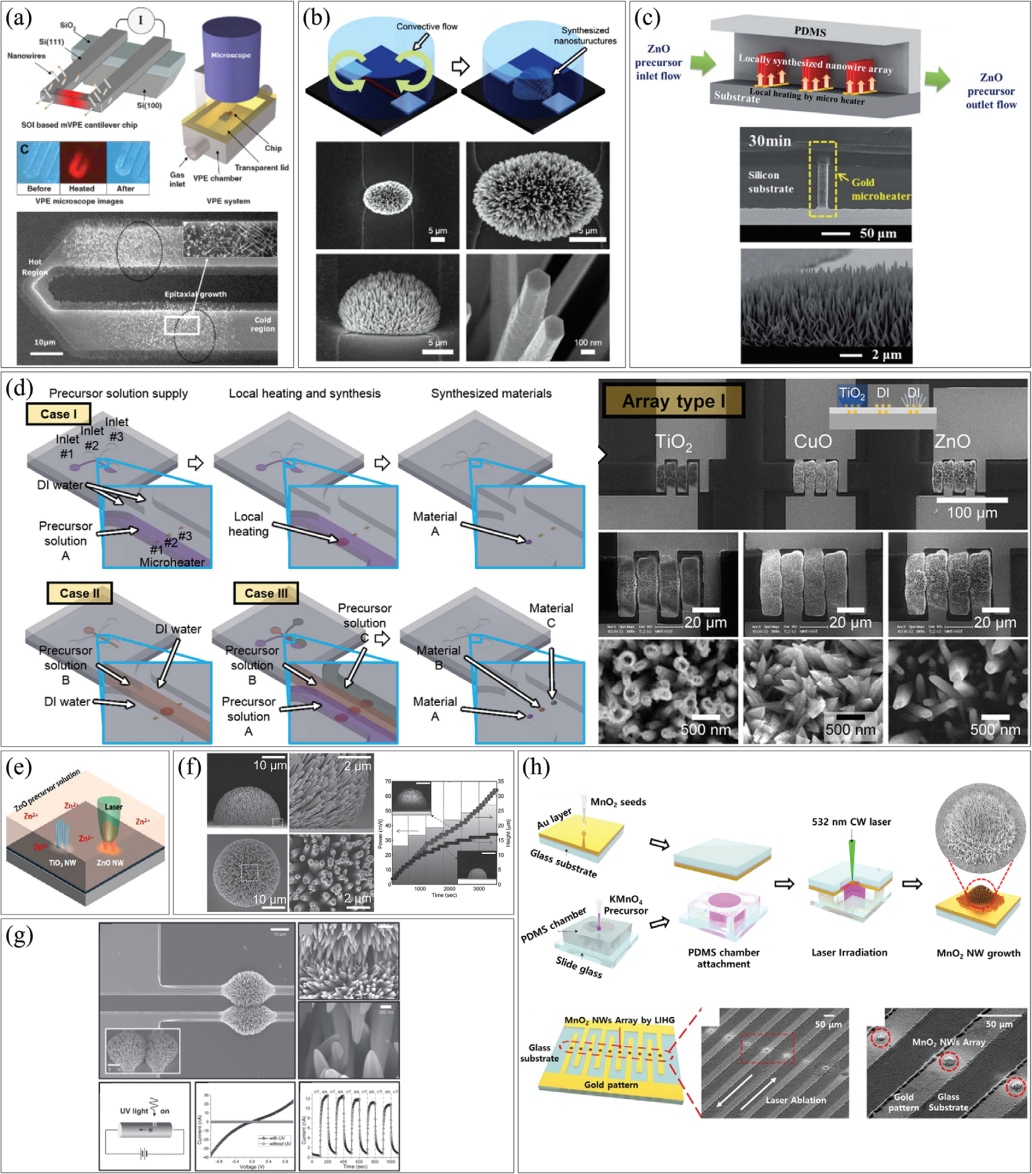
Direct synthesis and integration of 1D nanostructures by local Joule heating. a) (top) Schematic illustration of the principle and setup of micro vapor‐phase epitaxy (µVPE), and (bottom) SEM image of SiNWs grown by µVPE. Reproduced with permission.^[^ ^101^ ^]^ Copyright 2008, Wiley‐VCH Verlag GmbH & Co. KGaA, Weinheim. b) (top) Schematic illustration of the focused‐energy‐field (FEF) synthesis mechanism, and (bottom) SEM images of locally synthesized ZnO NWs. Reproduced with permission.^[^ ^91^ ^]^ Copyright 2014, Wiley‐VCH Verlag GmbH & Co. KGaA, Weinheim. c) (top) Schematic illustration of the FEF synthesis of NWs in a microfluidic channel, and (bottom) SEM images of ZnO NW bundles. Reproduced with permission.^[^ ^102^ ^]^ Copyright 2011, Royal Society of Chemistry. d) (left) Schematic illustration of the microfluidics‐based fabrication of heterogeneous nanomaterial arrays, and (right) SEM images of heterogeneous nanomaterial arrays consisting of TiO_2_ NTs, CuO nanospikes and ZnO NWs. Reproduced with permission.^[^ ^103^ ^]^ Copyright 2015, Springer Nature Limited. Direct synthesis and integration of 1D nanostructures by local laser‐induced heating. e) Schematic illustration of the laser‐induced hydrothermal growth (LIHG). Reproduced with permission.^[^ ^105^ ^]^ Copyright 2015, American Chemical Society, f) (left) SEM images, and (right) the growing kinetics of ZnO NWs synthesized by LIHG. Reproduced with permission.^[^ ^106^ ^]^ Copyright 2013, Wiley‐VCH Verlag GmbH & Co. KGaA, Weinheim. g) (top) SEM images of ZnO NWs synthesized by LIHG on parallel metal electrodes, and (bottom) their UV sensing application. Reproduced with permission.^[^ ^107^ ^]^ Copyright 2013, Wiley‐VCH Verlag GmbH & Co. KGaA, Weinheim. h) (top) Schematic illustration of MnO_2_ NWs synthesis process by LIHG, and (bottom) SEM images of MnO_2_ NWs on Au electrodes. Reproduced with permission.^[^ ^108^ ^]^ Copyright 2021, Elsevier.

Englander et al. and several other groups have achieved selective growth of CNTs and SiNWs at desired locations through the CVD process with well‐controlled localized Joule heating of microheaters.^[^ ^99^, ^100^, ^101^ ^]^ The microheater device was placed in a vacuum chamber and connected to an external power supply. The heating power was adjusted to reach the appropriate temperature for synthesizing the NWs. As shown in **Figure** **13**a, the NWs were only synthesized in the optimal temperature region, avoiding regions that were either too hot or too cold.^[^ ^101^ ^]^ Yang et al. reported localized hydrothermal synthesis achieved through local Joule heating using three different heater platforms: a Si microheater on a silicon‐on‐insulator (SOI) substrate, an Au microheater on a polyimide (PI) substrate, and multiple‐layered metal microheater/electrodes on a Si wafer (Figure 13b).^[^ ^91^ ^]^ The localized temperature rise of these microheaters in the precursor solution induced convective heat and mass transfers, resulting in the synthesis of nanomaterials at local hot spots through endothermic reaction of precursor chemicals. The synthesis temperature was as low as 90–95 °C, and the entire process could be conducted under atmospheric pressure conditions. The locally synthesized ZnO NWs on flexible devices exhibited excellent mechanical robustness due to direct synthesis on the electrodes. Furthermore, the study demonstrated various applications in microelectronic devices, including UV photodetectors and chemical sensors. Kim et al. were the first to demonstrate the synthesis of NWs through microscale temperature control while supplying precursors through a microchannel.^[^ ^102^ ^]^ They applied 2.0 × 10^15^, 2.6 × 10^15^, and 3.0 × 10^15^ W m^−3^ of power to the microheater to generate heat by Joule heating. The length and diameter of the NWs were increased by increasing the power. They also analyzed the size of the NWs by position. The NWs on the upstream side were larger than those on the downstream side, which they attributed to the fresh precursor solution being fed to the upstream side. They also showed the potential of ZnO NW arrays fabricated through this method for applications in particle trapping and pH level sensing, which can be utilized in biochemistry and single‐cell studies (Figure 13c). Building upon this approach, Yang et al. employed multiple microheaters for individual heating control and microfluidic channels for the selective supply of different precursor solutions along the laminar flow stream (Figure 13d).^[^ ^103^ ^]^ They achieved the synthesis of three different nanomaterials at desired locations by supplying their respective precursor solutions to three individual inlets and one common channel where they merge, while simultaneously generating Joule heating in all three microheaters. In doing so, they found the appropriate fluidic conditions (i.e., Reynolds number) through numerical simulation to minimize the cross‐contamination between different reaction regions caused by diffusion or convection of precursor solutions. Under these optimized fluidic conditions, they successfully synthesized titanium dioxide (TiO_2_) NTs, CuO nanospikes, and ZnO NWs by programmable heating the individual microheaters on a single device. This fabricated device was employed for multiplexed gas sensing, utilizing sensor arrays composed of different nanomaterials, which enabled enhanced selective gas detection.^[^ ^103^, ^104^ ^]^

#### 3.2.2 Laser‐Induced Heating

Yeo et al. introduced a laser‐induced local heating method for the digital local synthesis of NWs without the need for fabricating microheaters (Figure 13e).^[^ ^105^ ^]^ This approach involved generating a local hot spot on a target substrate through localized photothermal reaction with focused laser irradiation. ZnO NWs were synthesized using laser‐induced hydrothermal growth (LIHG), and their position could be easily adjusted by changing the location of the focused laser spot. As shown in Figure 13f, the cluster exhibited a pseudo‐hemispherical shape based on the temperature distribution.^[^ ^106^ ^]^ These NW clusters formed an electrical channel between two electrodes, which was utilized for UV sensing applications (Figure 13g).^[^ ^107^ ^]^ Additionally, manganese dioxide (MnO_2_) NWs, which typically require high temperature and high‐pressure conditions in an autoclave, were successfully synthesized using a low energy of 48 mW (Figure 13h).^[^ ^108^ ^]^

These local heating methods only heat small areas, and they provide notable advantages when compared to conventional global heating methods. These benefits include reduced power consumption, shorter synthesis time, and lower cost. Furthermore, by enabling control over the reaction conditions at each synthesis site, it is possible to adjust shapes and compositions at specific spots. Additionally, these methods address the issue of variations between the central region and the edges that often occur during global heating, resulting in more uniform nanodevices.

## 4 Functional Devices Using Integrated NWs

In Chapter 3, we explored various techniques for integrating NWs and provided concise explanations of their applications. Extensive research has focused on the exceptional mechanical, electrical, and optical properties of NWs, which have enabled their substitution for bulk materials in various fields such as electronic devices, energy harvesters, sensors, and more. In order to illustrate the superiority and practicality of integrated NWs, we would like to present a curated selection of papers that highlight their capabilities.

### 4.1 Electrodes

**Figure 14:**
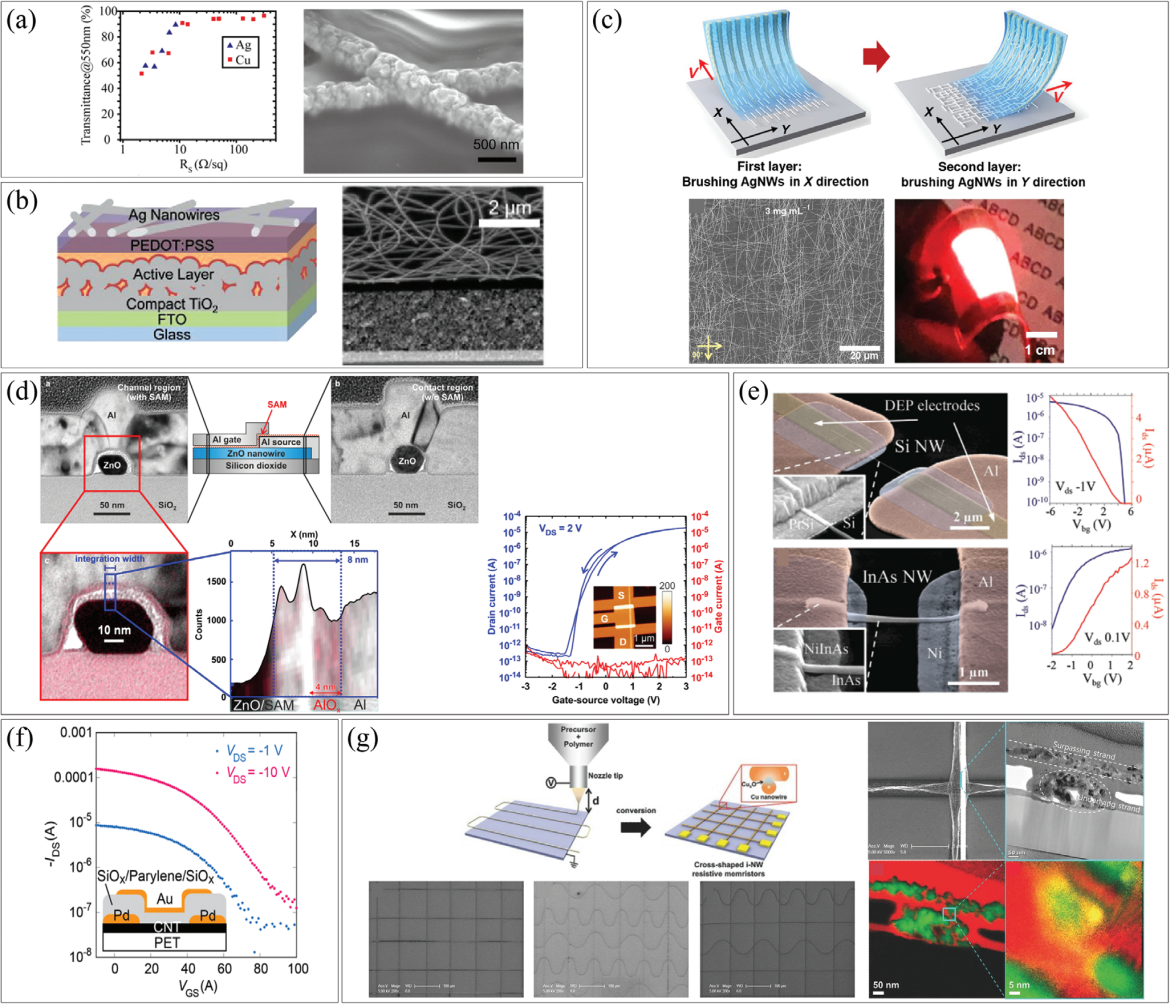
Applications of nanostructure integrated devices to transparent electrodes. a) (left) *R*_s_−*T* performance of electroless‐deposited NW transparent electrodes, and (right) SEM image of the AgNW junction. Reproduced with permission.^[^ ^47^ ^]^ Copyright 2014, American Chemical Society. b) (left) Schematic illustration of semitransparent solid‐state dye‐sensitized solar cells (ssDSCs), and (right) SEM image of the ssDSC. Reproduced with permission.^[^ ^109^ ^]^ Copyright 2013, Wiley‐VCH Verlag GmbH & Co. KGaA, Weinheim. c) (top) Schematic illustration of transparent electrode fabrication process using the writing and brushing method, (bottom left) SEM image of the double‐written electrode, and (bottom right) photo of T‐QLED emitting light. Reproduced with permission.^[^ ^110^ ^]^ Copyright 2020, Chinese Chemical Society. Applications of nanostructure integrated devices to transistors. d) (left) TEM analysis of transistor cross sections and, (right) transfer and output characteristics of a ZnO NW transistor with an Al top‐gate electrode, and (inset) AFM image of the transistor. Reproduced with permission.^[^ ^111^ ^]^ Copyright 2014, American Chemical Society. e) (left) SEM images and (right) *I*_DS_−*V*_S_ characteristics of (right top) Si p‐FET and (right bottom) InAs n‐FET. Reproduced with permission.^[^ ^61^ ^]^ Copyright 2014, Wiley‐VCH Verlag GmbH & Co. KGaA, Weinheim. f) Transfer characteristic curves obtained from a transistor made with roll‐to‐roll assembled SWCNT networks. Reproduced with permission.^[^ ^112^ ^]^ Copyright 2014, American Chemical Society. g) (left top) Schematic illustration of the fabrication process of memristor using EHD printing, (left bottom) SEM images of memristor array, and (right) electron energy loss spectroscopy (EELS) mapping images at the junction of the memristor. Reproduced with permission.^[^ ^113^ ^]^ Copyright 2015, WILEY‐VCH Verlag GmbH & Co. KGaA, Weinheim.

Owing to the excellent optical transparency and high electrical conductivity, metal NWs and metal NW composites have found wide‐ranging applications as current collectors or electrodes in energy harvesting devices, such as photovoltaic cells,^[^ ^32^, ^51^, ^52^ ^]^ and optical devices, including displays, smart windows, touchscreens, and more.^[^ ^33^, ^49^, ^76^ ^]^ Hsu et al. demonstrated the use of electrospun Ag/Cu NW networks with remarkable properties, exhibiting a high transmittance of 91% and a low sheet resistance of 8.5 Ω sq^−1^.^[^ ^47^ ^]^ The sheet resistance–transmittance (*R*s–*T*) relationship of the electrolessly deposited metal NWs showcased superior performance compared to conventional chemically synthesized metal NW networks (**Figure** **14**a). This improvement could be attributed to the electrospinning method, which produces NWs that are significantly longer (100 to 1000 times) than their chemically synthesized counterparts, resulting in lower junction resistance. Furthermore, during the metallization process, the junctions of metalized NWs naturally fused together, effectively reducing high junction resistance. This electroless method holds considerable promise for large‐area optoelectronic devices, such as photovoltaics and LEDs. Margulis et al. introduced a spray deposition method for AgNWs and their application in solid‐state dye‐sensitized photovoltaic cells (ssDSC) (Figure 14b).^[^ ^109^ ^]^ The device structure and fabrication procedure closely resembled that of a standard ssDSC, with the exception of the top electrode. Instead of conventional electrodes, AgNWs dispersed in methanol solution were spray‐coated onto the device, resulting in a sparse, uniform mesh with well‐dispersed wires and wire‐to‐wire gaps in the range of 1–2 µm. The transmittance of the spray‐deposited AgNWs exceeded 90% between wavelengths of 450 and 700 nm. The as‐sprayed AgNWs exhibited a very low sheet resistance of 18 Ω sq^−1^ without requiring any post‐treatment processes. The fabricated photovoltaic cells achieved a competitive energy conversion efficiency of 3.6%. Meng et al. employed AgNWs dispersed in isopropyl alcohol (IPA) and aligned the NWs using a brushing process on a PET substrate (Figure 14c).^[^ ^110^ ^]^ This writing and brushing process was repeated in a perpendicular direction, resulting in a cross‐linked NW mesh. These flexible transparent electrodes (FTEs) exhibited a high transmittance of 93.8% and a sheet resistance of 21.4 Ω sq^−1^, while maintaining good stability against bending. Leveraging these characteristics, the authors applied the FTEs to transparent quantum dot light‐emitting diodes (T‐QLEDs) with a high external quantum efficiency (EQE) of 15.47%.

Since expensive methods are not suitable for fabricating electrodes, methods that can easily deposit on a large area such as electrospinning, spray coating, and dispersion are used. Despite using a simple method, as mentioned in these examples, low electrical resistance was implemented for the use of electrodes, and optical applications were feasible due to the smaller diameter than the wavelength of light.

### 4.2 Transistors

Semiconducting NW‐based transistors have attracted considerable attention due to their ability to utilize a variety of materials, including CNTs and compound semiconductors, and their potential for miniaturization. The integration methods described above present novel and innovative approaches to fabricating transistors, complementing the traditional CMOS process.

Kälblein et al. reported a high‐performance ZnO NW‐based transistor.^[^ ^111^ ^]^ Pre‐synthesized and annealed ZnO NWs were randomly dispersed on a SiO_2_/Si substrate. Source, gate, and drain contacts were fabricated using e‐beam lithography and aluminum (Al) evaporation. To improve insulation and enhance performance, an octadecylphosphonic acid‐based SAM was formed on the substrate, creating a hybrid organic/inorganic (SAM/AlO_x_) gate dielectric with an ultra‐small thickness and a gate‐all‐around structure. The transfer and output characteristics of a ZnO NW transistor with an Al top‐gate electrode and a 1 µm channel length are shown in the graph in Figure 14d. The transistor exhibited excellent performance, including a 10^7^ on/off current ratio, 150 mV per decade subthreshold slope, and a peak transconductance of under 7 µS. Notably, the gate current of the transistor remained below 0.1 pA across the entire gate‐source voltage range. Additionally, this hybrid gate dielectric enabled compatibility with organic LEDs, as demonstrated by the authors through the application of switching and driving organic LEDs. Collet et al. introduced a capillary‐assisted dielectrophoresis method for large‐scale assembly of NWs, enabling precise positioning and alignment of single Si NWs or InAs NWs onto desired source‐drain electrodes.^[^ ^61^ ^]^ This method facilitated the fabrication of Si p‐FET and InAs n‐FET devices (Figure 14e), demonstrating its capability for large‐scale and multiple‐material assembly. Kiriya et al. achieved the assembly of single‐walled carbon nanotubes (SWCNTs) on flexible substrates using a selective roll‐to‐roll process.^[^ ^112^ ^]^ By coating SWCNTs on a PET film based on surfactant‐substrate interactions, they obtained a high‐density SWCNT film with good coverage. It served as a channel for thin‐film transistors (TFTs) and exhibited satisfactory performance, as shown in Figure 14f. Xu et al. presented a facile method for fabricating memristors using the EHD printing process (Figure 14g).^[^ ^113^ ^]^ They electrohydrodynamically printed a solution containing polyvinylpyrrolidone (PVP) and copper trifluoroacetate (CTA) onto a substrate. Subsequently, Cu_x_O NWs were obtained through a heating process, where the Cu_x_O NWs were then reduced back to CuNWs in a high‐temperature hydrogen atmosphere. Most of the Cu_x_O was reduced to Cu, while some remained as Cu_x_O at the intersections. These remaining Cu_x_O acted as memristors, and CuNWs acted as electrodes.

In this section, we have shown examples of pre‐fabricated NWs assembling to a substrate and acting as transistors. While these devices perform reasonably well, they do not perform as well as transistors that have been sophisticatedly fabricated in the CMOS process. However, they have advantages over the CMOS process‐based materials in controlling the composition, crystallinity, and shape of NWs, and therefore have their own unique applications.

### 4.3 Energy Harvesters

The piezoelectric effect refers to the generation of electricity through the polarization of a material with a specific crystal structure when subjected to deformation.^[^ ^114^ ^]^ The small size of nanostructures, particularly NWs, make them efficient for piezoelectric power generation, as even minor deformation can be converted into substantial strain and stress.

**Figure 15:**
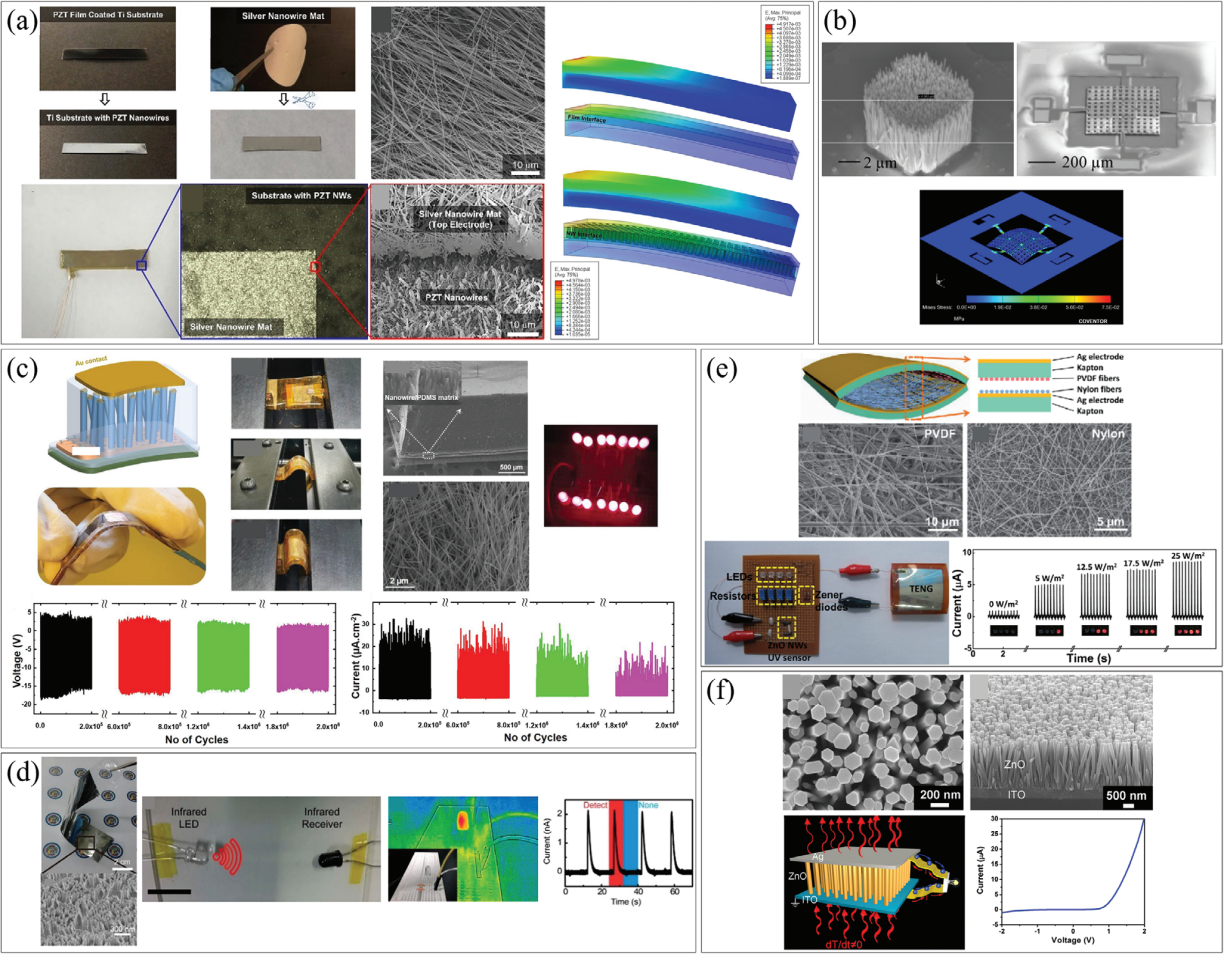
Applications of nanostructure integrated devices to energy harvesters. a) (left) Photo and SEM images of a cantilever‐type energy harvester consisting of vertically aligned PZT NWs, and (right) numerical simulation of energy harvesting result. Reproduced with permission.^[^ ^115^ ^]^ Copyright 2018, Elsevier. b) (top) SEM images of membrane/proof‐mass structure energy harvester consisting of vertically grown ZnO NWs, and (bottom) numerical simulation of energy harvesting result. Reproduced with permission.^[^ ^116^ ^]^ Copyright 2016, Elsevier. c) (top) Schematic illustration, photo and SEM images of a GaN NW‐based nanogenerator, and (bottom) its long‐term stable power generation performance. Reproduced with permission.^[^ ^117^ ^]^ Copyright 2020, Wiley‐VCH GmbH. d) (left) Photo and SEM images of the TENG device consisting of FEP NW, (middle) photograph of the infrared LED powered by TENG and infrared image during operation, and (right) the current of the infrared receiver when detecting the infrared signals. Reproduced with permission.^[^ ^119^ ^]^ Copyright 2017, AIP Publishing. e) (top) Schematic illustration of the TENG device consisting PVDF and nylon NWs, (middle) SEM images of PVDF and nylon NWs, and (bottom) experimental setup of self‐powered UV detection system driven by TENG and the current across UV sensor. Reproduced with permission.^[^ ^120^ ^]^ Copyright 2014, Royal Society of Chemistry. f) (top) SEM images of vertically grown ZnO NWs for PyNG, (bottom left) Schematic illustration of the PyNG, and (bottom right) *I*−*V* characteristics of the PyNG. Reproduced with permission.^[^ ^122^ ^]^ Copyright 2012, American Chemical Society.

Malakooti et al. fabricated a piezoelectric nanogenerator (PENG) using lead zirconate titanate (PZT) NWs, with dimensions of 30 mm length, 8 mm width, and 1 µm height, through a hydrothermal reaction (**Figure** **15**a).^[^ ^115^ ^]^ They compared the power generation of PZT films with and without NWs under various vibration conditions. The device incorporating NWs allowed large deformation and the creation of a functional gradient, blending the mechanical properties of two dissimilar materials. This resulted in a 7.2‐fold increase in power generation (187 nW under 1 g root mean square (RMS) acceleration) compared to the bulk film. Li et al. selectively synthesized an 8 × 8 µm^2^ ZnO NW block using pulsed laser deposition (PLD) and the hydrothermal synthesis method (Figure 15b).^[^ ^116^ ^]^ The NW block was topped with a silicon nitride (Si_3_N_4_) membrane, and Ni was electroplated to serve as a proof mass. The ZnO NWs experienced compression and stretching by the Si_3_N_4_ membrane with Ni proof mass in response to external vibrations. This device achieved a power output of 70 pW (corresponding to 22 mW cm^−3^) under 1.4 g acceleration. The vertically aligned crystalline ZnO NWs led to this high efficiency under this low acceleration. Numerous studies have confirmed the superiority of nanomaterial‐based power generators. Additionally, durability is a crucial factor for practical use. Johar et al. developed a PENG using vertically grown GaN NWs and subjected the device to 4 million stretching‐releasing cycles to assess its durability (Figure 15c).^[^ ^117^ ^]^

The above three PENG devices were fabricated by pre‐patterned hydrothermal synthesis or epitaxial growth. These fabrication methods led to strong bonds between the substrate and NWs, which contributed to improved energy production efficiency and mechanical robustness of devices.

A triboelectric nanogenerator (TENG) is a device that utilizes the triboelectric effect to convert mechanical motion into electrical energy.^[^ ^118^ ^]^ Since friction is a surface action, a large surface area of nanomaterial is a major advantage. Additionally, polymeric materials typically have higher efficiencies, so it is important to fabricate polymeric nanodevices with a large surface area. Li et al. developed a TENG based on fluorinated ethylene propylene (FEP) (Figure 15d).^[^ ^119^ ^]^ They fabricated vertically aligned FEP NWs by a pattering and etching process. They aimed to harness energy from water waves, and the TENG they made produced a 10 µA and 200 V output, successfully powering an infrared LED in the process. The patterning and etching method used in this work can be applied to fabricate NWs from a variety of polymers, which is important for increasing material diversity. Zheng et al. developed a TENG by fabricating electrospun poly(vinylidene fluoride) (PVDF) and polyamide (nylon) fibers on PI film with Ag electrodes (Figure 15e).^[^ ^120^ ^]^ Electrospinning is a method that can produce dense NWs over a large area, making it a suitable method for producing TENG devices in which large surface areas are important, and it has shown the following outstanding results. The device achieved a peak power density of 26.6 W m^−2^ under a 5 Hz triggering frequency with a 20 mm amplitude, successfully powering a UV detector.

Nano‐piezoelectric and triboelectric materials have garnered significant attention in the development of energy harvesters, as they can harness energy from small natural movements such as wind, waves, or vibrations from machines.

The Pyroelectric nanogenerator (PyNG) is another energy harvesting device. It operates based on polarization, similar to the PENG, but achieves this effect by temperature fluctuations.^[^ ^121^ ^]^ Consequently, nanodevices with low heat capacity and high crystallinity are commonly utilized. In a study by Yang et al., they synthesized ZnO NWs with a diameter of 200 nm and a length of 2 µm on an ITO substrate using the hydrothermal method (Figure 15f).^[^ ^122^ ^]^ An Ag film was deposited to serve as an electrode. The PyNG had an approximate area of 15 mm^2^, and its efficiency in converting heat flow into electricity was estimated to be within the range of 0.05–0.08 Vm^2^ W^−1^. Hydrothermal synthesis is a method for synthesizing crystalline materials, such as ZnO, in one directional alignment. Therefore, it is a suitable method for fabricating these PyNG devices. It basically enables synthesis over a large area, and when combined with the methods introduced in Chapter 3, it also provides integration in a specific area.

### 4.4 Physical/Photonic Sensors

Assembled NW devices can also be utilized for sensing various physical stimuli, including pressure, strain, force, temperature, and light intensity. Pressure, force, and strain sensors are among the most common types of physical sensors. The NWs used in these sensors can be categorized into piezoelectric, resistive, and capacitive types based on their sensing mechanisms.

**Figure 16:**
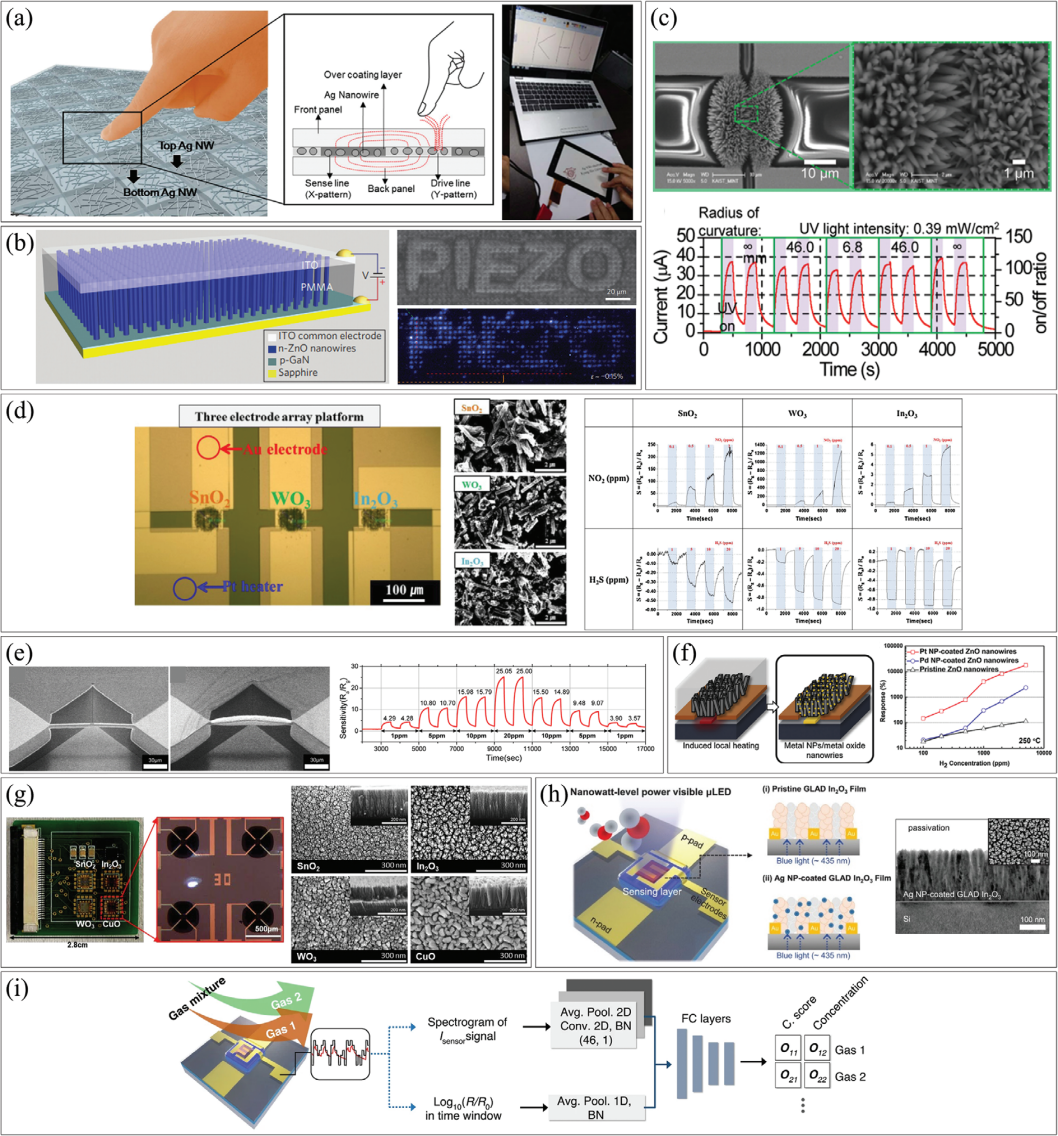
Applications of nanostructure integrated devices to physical and photonic sensors. a) (left) Schematic illustration and (middle) operation principle of flexible touch screen panels composed of roll‐to‐roll slot‐die coated AgNW network films, and (right) a picture of that in a real application. Reproduced with permission.^[^ ^53^ ^]^ Copyright 2016, Springer Nature Limited. b) (left) Schematic illustration of the NW‐LED‐based pressure sensor array, (right) optical image of the device with a convex mold on top and electroluminescence images of the device at strains of 0.15%. Reproduced with permission.^[^ ^123^ ^]^ Copyright 2013, Springer Nature Limited. c) (top) SEM images of ZnO NW‐based UV detector fabricated by localized hydrothermal synthesis, and (bottom) UV sensing results with various bending conditions. Reproduced with permission.^[^ ^124^ ^]^ Copyright 2018, American Chemical Society. Applications of nanostructure integrated devices to chemical sensors. d) (left) Microscopic image of gas sensor array consisting of heterogeneous nanofibers (SnO_2_, WO_3_ and In_2_O_3_) fabricated by EHD printing on the Au sensing electrodes and Pt microheaters, and (right) their sensing results for NO_2_ and H_2_S gases with a power of 20 mW per microheater. Reproduced with permission.^[^ ^77^ ^]^ Copyright 2017, Elsevier. e) (left) SEM images of locally synthesized SnO_2_ NTs on a freestanding microheater, and (right) their H_2_S sensing results. Reproduced with permission.^[^ ^125^ ^]^ Copyright 2017, American Chemical Society. f) (left) Schematic illustration of surface modified ZnO NWs with Pd and Pt NPs, and (right) their hydrogen sensing results. Reproduced with permission.^[^ ^126^ ^]^ Copyright 2015, Elsevier. g) (left) Photo of machine learning applied high‐performance gas sensor system based on locally deposited NW array, and (right) SEM images of sensing materials. Reproduced with permission.^[^ ^127^ ^]^ Copyright 2022, American Chemical Society. h) (left) Schematic illustrations of a blue µLED‐based gas sensor, and (right) Cross‐sectional TEM image and top‐view SEM image of nano‐porous Ag NPs‐coated In_2_O_3_ film. Reproduced with permission.^[^ ^129^ ^]^ Copyright 2023, Wiley‐VCH GmbH. i) Architecture of modified deep convolutional neural network for identifying gas mixtures. Reproduced with permission.^[^ ^130^ ^]^ Copyright 2023, Springer Nature Limited.

Kim et al. developed large‐area touchscreen panels (TSPs) as shown in **Figure** **16**a.^[^ ^53^ ^]^ Since low‐resistance thin films needed to be coated over a large area, the authors used roll‐to‐roll patterning process with AgNWs. They controlled the density of AgNWs by adjusting the injection flow rate of an AgNW ink. The long AgNWs provided low sheet resistance (30–70 Ω sq^−1^) and high optical transmittance (89–90%) for the capacitive‐type touch sensor. This fabrication process offers an alternative to high‐cost ITO films prepared through vacuum‐based sputtering processes. Pan et al. developed a high‐resolution pressure‐imaging device based on the piezoelectric and electroluminescent properties of ZnO NWs (Figure 16b).^[^ ^123^ ^]^ A patterning process and subsequent ZnO hydrothermal synthesis process were used to align the crystalline NWs in one direction over a relatively large area. These n‐type ZnO NWs were synthesized on a p‐type GaN substrate to act as light emitters. Applying vertical pressure to the ZnO NWs generated piezoelectric potentials within the crystal, leading to the flow of carriers between the n‐ZnO and p‐GaN and the emission of light due to electron–hole recombination. The size of the LEDs was the same as the diameter of the ZnO NWs, which was determined in the patterning process and, enabled ultra‐small spatial resolution (under 2.7 µm) for pressure mapping. Yang et al. fabricated a wavelength‐tunable light sensor on a flexible substrate using localized and direct hydrothermal synthesis of ZnO NWs and subsequent selective LPD process of TiO_2_ NTs.^[^ ^124^ ^]^ This ZnO/TiO_2_ hybrid material enabled the detection of light ranging from UV to the visible range. Because they only needed one spot for the device, they used a localized hydrothermal synthesis method using Joule heating from microheater rather than the more commonly used patterning process. Since the ZnO NWs were synthesized directly on the electrode via hydrothermal synthesis, which led robust connection between ZnO NWs and electrodes. Consequently, the devices exhibited reliable light sensing performance even under repeated mechanical bending conditions (6.8 mm radius of curvature and 1000 cycles), as shown in Figure 16c.

### 4.5 Chemical Sensors

Integrated NWs can be also used as chemical sensors. The high surface‐to‐volume ratio of nanomaterials enables a high response to gas molecules, and their sparse network allows for effective diffusion of gas molecules into the interspace of NWs. Combining NWs with MEMS heating platforms has enabled researchers to achieve high‐sensitivity sensing and low power consumption operation.

Kang et al. integrated various metal oxide NWs (SnO_2_, WO_3_, In_2_O_3,_ and nickel oxide (NiO)) on microheaters using EHD printing, as shown in Figure 16d.^[^ ^77^ ^]^ Electrospun NWs with granular nanostructures and rough surfaces enabled highly sensitive gas detection. The EHD printing method allowed the jetting of NW inks onto freestanding microheaters with an area of less than 100 × 100 µm^2^. This resulted in low operating power (<20 mW) owing to their small heat capacity. EHD printing proved advantageous for integrating multiple types of NWs on a single device without fabrication‐induced interferences. The researchers demonstrated the selective detection of different gas species (NO_2_ and H_2_S gas) with multiplexed gas sensors composed of three different metal oxide NWs (SnO_2_, WO_3,_ and In_2_O_3_) and their signal processing. Cho et al. achieved highly sensitive and low power‐consuming gas sensors by utilizing localized hydrothermal synthesis on a freestanding microheater.^[^ ^125^ ^]^ As mentioned earlier, Joule heating combined with hydrothermal synthesis is a useful method for synthesizing and integrating a material in a small area. ZnO NWs were directly synthesized on the Joule‐heated area (3 × 100 µm^2^). Additionally, the LPD process facilitated the formation of a porous SnO_2_ thin film on the surface of ZnO NWs and simultaneous etching of the ZnO core, resulting in porous SnO_2_ NTs with improved sensing performance, as depicted in Figure 16e. This method provided a downscaled integration area and achieved one of the lowest power‐consuming gas sensors (<6 mW) among metal oxide‐based chemiresistive gas sensors. Surface modification with catalytic noble metals is a key strategy for developing high‐performance chemical sensors. Kim et al. succeeded in directly synthesizing the ZnO NWs decorated with Pd/platinum (Pt) NPs through sequential localized hydrothermal reactions.^[^ ^126^ ^]^ First, ZnO NWs were grown on a micro‐heating device through local Joule heating. Subsequently, the ZnO NWs immersed in a metal precursor were heated again using the embedded microheater. The heat conducted through the NWs raised the surface temperature, leading to in situ generation of Pd or Pt NPs on the surface, as shown in Figure 16f. The catalytic effect of the NPs, such as spillover on the surface, significantly improved the sensing response and recovery time for hydrogen gas, even at low operating temperatures of 100 °C. Furthermore, Kang et al. developed an advanced gas mixture sensing device, as depicted in Figure 16g.^[^ ^127^ ^]^ The sensing materials array consisted of SnO_2_, In_2_O_3_, WO_3_, and CuO nanopillars, deposited using the glancing angle deposition (GLAD). This is a specialized type of PVD method that utilizes large glancing angle and rotating substrate to form NWs.^[^ ^128^ ^]^ It can also be combined with the patterning process to adjust the area and position of the device, and provides a strong bond to the substrate by growing from the surface of the substrate. Furthermore, as shown in Figure 16h, Cho et al. utilized the GLAD to integrate In_2_O_3_ onto micro‐LED and developed a gas sensor that operates at an extremely low power of only 63 nW.^[^ ^129^ ^]^ Recently, these advanced material integration methods and data analysis and processing technologies such as convolutional neural network (CNN) are driving more sensitive, accurate, and rapid gas sensor system (Figure 16i).^[^ ^127^, ^130^, ^131^ ^]^

## 5 Conclusion

In this article, we discussed the up‐to‐date methods for integrating 1D nanomaterials for functional devices. Over the past decade, pre‐synthesis and post‐assembly methods such as random dispersion, masking and lift‐off, inkjet printing, electrospinning, contact printing, roll‐to‐roll printing, atomic force microscopy‐based manipulation, fluid drag force assembly, capillary force assembly, dielectrophoresis assembly, magnetic field assembly, electrohydrodynamic printing, optical tweezer, Langmuir‐Blodgett technique, and substrate stretching have remained the mainstream approaches for nanomaterial integration. Each method has its own advantages and limitations in terms of accuracy and mass production, and their suitability is often dependent on the properties of the materials involved. These methods have become more sophisticated and are being applied to produce high‐performance devices. The most significant advancement in nanodevice fabrication over the past decade is the development of direct synthesis and integration methods. In these techniques, local hotspots are generated through Joule heating or laser‐induced heating, and nanostructures are synthesized at desired locations. These methods enable the direct synthesis and simultaneous integration of functional nanomaterials, simplifying the fabrication process of nanodevices.

We have introduced over a dozen methods for 1D nanomaterial assembly. Regardless of whether NWs are being aligned in a large or small area, the major challenge in assembly is achieving uniform alignment. Many researchers have studied methods for achieving uniform and accurate alignment, but these methods inevitably involve expensive, complex, and time‐consuming processes. It is necessary to continuously develop innovative assembly methods, but it is equally important to obtain more uniform NWs during the NW synthesis and harvesting processes. Another key issue is the robust contact between nanomaterials and substrates. Since most assembly methods do not involve additional bonding processes, the attachment between the nanomaterials and substrate (or electrodes) relies on van der Waals forces. For stronger mechanical bonding or specific chemical bonding, a metal is often deposited at the junctions, or the surface is modified in advance. However, these processes require additional steps that are as complex as the assembly process itself. In the case of direct integration methods, since NWs are synthesized directly on the surface, reliable mechanical and electrical bonding is guaranteed, but controlling their strength or chemical bonds remains challenging. Research on the analysis and control of these junctions is also essential for the fabrication of superior NW‐based devices.

It is evident that nanomaterials can provide unique properties that are not present in bulk materials, owing to their quantum effects, dimensions below the mean free paths, high surface energy, low density of defect, grain size effects, and extremely high surface‐to‐volume ratio. Despite these properties having been discovered for a long time period, the primary obstacle to the widespread commercialization of nanomaterial‐based devices is believed to be the lack of advanced assembly and integration techniques. This review is expected to stimulate interest in the fabrication technology for nanodevices, provide an overview of recent advancements and the evolution of 1D nanomaterial integration methods, and potentially offer guidance for future research directions.

## Conflict of Interest

The authors declare no conflict of interest.
